# From Food Choice to Nutrigenomic Response: Integrating Nutrigenetics and Nutrigenomics Toward Precision Nutrition

**DOI:** 10.3390/nu18213486

**Published:** 2026-10-23

**Authors:** Salvatore Saccone, Francesca Bruno, Desiree Brancato, Elvira Coniglio, Valentina Sturiale, Concetta Federico

**Affiliations:** 1 Department of Biological, Geological and Environmental Sciences, University of Catania, 95124 Catania, Italy; francesca.bruno@unikore.it (F.B.); desiree.brancato@unict.it (D.B.); elvira.coniglio@phd.unict.it (E.C.); valentina.sturiale@phd.unict.it (V.S.); concetta.federico@unict.it (C.F.); 2 Department of Medicine and Surgery, Kore University of Enna, 94100 Enna, Italy

**Keywords:** nutrigenetics, nutrigenomics, precision nutrition, gene–diet interaction, genetic variants, dietary exposure, epigenetics, gut microbiota, obesity, clinical actionability

## Abstract

Nutrigenetics and nutrigenomics represent complementary dimensions of the complex relationship between diet and the human genome and provide a conceptual basis for precision nutrition. Nutrigenetics investigates how interindividual genetic variability influences food perception, dietary behavior, nutrient metabolism, and biological responses, whereas nutrigenomics explores how nutrients and bioactive food components modulate gene expression, signaling pathways, and epigenetic mechanisms. Importantly, these processes are interconnected: genetic variation may influence dietary exposure itself through effects on sensory perception, food preferences, appetite, and eating behavior, while additional genetic differences can modify nutrient absorption, metabolism, bioavailability, and downstream molecular responses. This review integrates these interactions within a diet–genome–phenotype framework, incorporating nutrient-responsive molecular pathways, epigenetic regulation, and the gut microbiota as additional determinants of interindividual variability. Overweight and obesity are examined as a paradigmatic application of this framework, illustrating how genetic determinants of dietary exposure and metabolic response may jointly contribute to susceptibility and heterogeneous responses to nutritional interventions. We further propose a research-oriented Weighted Evidence Score (WES) for the evidence-based prioritization of candidate genetic variants, integrating seven domains of functional evidence, phenotypic evidence, independent replication, genotype–diet interactions, nutritional intervention evidence, incremental predictive value, and population validity. In a pilot application to twelve illustrative variants or haplotypes, eight were classified as Tier 2 and four as Tier 3, with none meeting the requirements for Tier 1. Rather than representing an immediately actionable clinical test, such a research-oriented panel is intended as a dynamic hypothesis-generating and validation tool integrating genotype with dietary exposure, metabolic and molecular biomarkers, lifestyle factors, and phenotypic outcomes. Particular emphasis is placed on distinguishing evidence-based research prioritization from the additional validation required for clinical actionability. Finally, ethical, professional, and social challenges related to genomic information, including informed consent, privacy, communication, population diversity, and equitable implementation, are considered. Precision nutrition should ultimately be defined not by the amount of molecular information collected, but by its demonstrated ability to provide incremental predictive value and improve nutritional interventions beyond what can be achieved using established clinical, dietary, and population-based approaches.

## 1. Introduction

Nutrigenomics and nutrigenetics represent complementary approaches to the study of diet–genome interactions and constitute two fundamental components of the emerging framework of precision nutrition [1,2,3]. Nutrigenomics investigates how nutrients and bioactive food compounds influence gene expression, cellular signaling pathways, and epigenetic mechanisms, whereas nutrigenetics examines how interindividual genetic variability affects food-related traits, nutrient absorption and metabolism, and biological responses to dietary components. Their integration is therefore essential for understanding why individuals exposed to comparable dietary conditions may exhibit markedly different molecular, metabolic, and phenotypic responses.

The relationship between diet and genome is inherently bidirectional. On the one hand, dietary components can act as metabolic substrates, signaling molecules, or modulators of transcriptional and epigenetic processes, thereby influencing cellular functions and ultimately contributing to phenotype. On the other hand, genetic variation can affect multiple processes that precede or modulate these molecular responses, including sensory perception, food preferences, nutrient intake, intestinal absorption, transport, enzymatic metabolism, and downstream signaling [4,5,6]. Thus, genetic variability may act both upstream of dietary exposure, by influencing what and how much is consumed, and downstream of dietary exposure, by modulating the biological response to nutrients and bioactive compounds.

This distinction becomes particularly relevant when moving from population-based nutritional recommendations toward precision nutrition. The biological activity of a dietary compound depends on its actual consumption, bioavailability, and interaction with molecular targets. Environmental, cultural, behavioral, and microbial factors further contribute to interindividual variability, making the resulting phenotype the product of interconnected genetic, nutritional, and environmental influences rather than a single determinant [5,7].

Accordingly, precision nutrition cannot be reduced to isolated gene–nutrient associations or the direct translation of individual genetic variants into dietary recommendations. It requires an integrated assessment of genetic variability, dietary exposure, molecular responses, and measurable phenotypic outcomes [5]. Nutrigenetics and nutrigenomics should therefore be considered complementary and interconnected approaches rather than separate disciplines.

In this review, we examine the molecular and genetic determinants underlying this reciprocal relationship, focusing first on genetic influences on food perception and dietary exposure and subsequently on nutrient-responsive molecular pathways, epigenetic regulation, and the contribution of the gut microbiota. We then integrate these components into a diet–genome–phenotype framework and consider overweight and obesity as a paradigmatic setting in which to explore its application to precision nutrition. Within this context, we discuss the rationale for a research-oriented panel of candidate genetic variants and propose an evidence-based framework for their evaluation. Finally, we critically examine the transition from gene–diet association to prediction and actionability, together with the ethical and social implications associated with the increasing use of genomic information in precision nutrition.

The methodological approach adopted in this review comprises two complementary components: a narrative synthesis of the broader nutrigenetics and nutrigenomics literature and a structured, variant-specific appraisal of the evidence supporting the proposed research-oriented genetic panel. The latter approach, including the literature assessment strategy and evidence-grading procedures, is described in detail in Section 8.7.

## 2. Genetic Determinants of Food Perception, Preference, and Dietary Exposure

Dietary exposure depends not only on food availability and conscious choice but also on sensory perception, food preferences, appetite, and eating behavior. These traits exhibit substantial interindividual variability arising from genetic, environmental, cultural, and behavioral influences [6]. Genetic differences may therefore contribute to variation in the consumption of specific nutrients and bioactive compounds, influencing the dietary exposures that precede nutrigenomic responses.

### 2.1. Genetic Variability in Taste Perception

Taste perception represents one of the best-characterized examples of genetic modulation of food-related traits. The major taste modalities have distinct physiological and evolutionary functions [8]:

- Sweetness, generally associated with the detection of energy-rich nutrients, particularly sugars;
- Umami, associated with amino acids and protein-rich foods;
- Saltiness, contributing to sodium sensing;
- Sourness, providing information on food acidity;
- Bitterness, traditionally associated with the detection of potentially harmful compounds.

In addition, oral perception of dietary lipids has emerged as an important sensory component of food selection, although its classification as an independent primary taste modality remains debated [9,10].

Among the most extensively investigated genetic determinants of taste perception is *TAS2R38*, encoding a G protein-coupled bitter taste receptor. Three common coding variants—rs713598, rs1726866, and rs10246939—define the major PAV and AVI haplotypes and account for a substantial proportion of interindividual variation in sensitivity to the bitter compounds phenylthiocarbamide (PTC) and 6-n-propylthiouracil (PROP). Individuals homozygous for the PAV haplotype generally exhibit greater sensitivity to these compounds than individuals homozygous for AVI, whereas heterozygous individuals typically show an intermediate phenotype [6,11].

The potential influence of *TAS2R38* variation extends to the perception of naturally bitter foods, including vegetables containing bitter phytochemicals. However, evidence linking *TAS2R38* genotype directly to habitual intake of cruciferous or other bitter vegetables is less consistent than that linking the gene to PTC/PROP perception. This distinction is important because sensory phenotype does not necessarily translate directly into dietary behavior, which is influenced by multiple environmental and experiential factors [6].

Genetic variability also contributes to individual differences in sweet taste perception. Sweet compounds are detected primarily through the heterodimeric receptor formed by taste receptor type 1 member 2 (*TAS1R2*) and taste receptor type 1 member 3 (*TAS1R3*). Polymorphisms in the corresponding genes have been investigated in relation to sweet taste sensitivity, preference for sweet foods, and sugar intake. Variation in *TAS1R2*, for example, has been associated with differences in sucrose perception and sugar consumption [12]. As with bitter taste, however, receptor genotype represents only one component of a complex phenotype influenced by previous dietary exposure, age, physiological status, and environmental and cultural factors.

### 2.2. Genetic Determinants of Oral Fat Perception

Dietary lipids provide an additional example of how genetic variation may influence food perception and, potentially, dietary exposure. Cluster of differentiation 36 (CD36) is a membrane glycoprotein with multiple biological functions, including fatty-acid binding and transport, and is expressed in taste receptor cells, where it contributes to the oral detection of long-chain fatty acids. The common *CD36* variant rs1761667 has been associated with interindividual differences in oral fat sensitivity, with experimental studies showing genotype-dependent differences in fatty-acid detection thresholds [13]. Subsequent studies have further investigated associations between *CD36* variants, fat perception, dietary characteristics, and metabolic phenotypes, although findings regarding food preference and habitual intake have not been fully consistent [14,15].

Reduced oral sensitivity to fatty acids could theoretically influence the concentrations required to generate comparable sensory perception and thereby contribute to differences in food selection. Nevertheless, the relationship among *CD36* genotype, sensory phenotype, dietary behavior, and metabolic outcomes remains complex and is influenced by multiple environmental and physiological factors. *CD36* therefore illustrates both the potential relevance and the limitations of extrapolating from a genetically influenced sensory phenotype to habitual dietary behavior.

### 2.3. From Sensory Genotype to Food Preference and Dietary Exposure

The biological relevance of taste-related genetic variation potentially extends beyond sensory perception itself. Differences in the perception of bitterness, sweetness, or dietary fat may influence food acceptance and habitual dietary choices and, consequently, the amount and frequency of exposure to specific nutrients and bioactive compounds. This introduces an important intermediate level into the relationship between genotype and nutrigenomic response:

**genetic variation** → **sensory phenotype** → **food preference** → **dietary exposure**

This sequence should not be interpreted as a deterministic pathway. Genetic predisposition operates within a complex environmental context. Food availability, family habits, cultural traditions, socioeconomic conditions, previous exposure, learning, and individual experience can reinforce, attenuate, or override genetically influenced sensory preferences. Accordingly, similar taste-related genotypes may be associated with different dietary behaviors in different individuals or populations [4,6].

For precision nutrition, genetically influenced sensory differences may affect exposure to dietary bioactive compounds, although actual intake also depends on food selection and consumption, while subsequent systemic exposure is further influenced by bioavailability. Sensory genotype alone is therefore insufficient to predict nutrigenomic responses.

A useful example is provided by the relationship between bitter taste perception and cruciferous vegetables. *TAS2R38* variation can influence the perceived bitterness of glucosinolate-containing vegetables, whereas its influence on their habitual consumption appears more variable across studies [6]. Cruciferous vegetables are dietary sources of glucosinolates from which biologically active isothiocyanates, including sulforaphane, can be generated [16]. Thus, genetically influenced sensory perception may represent one of several factors contributing to differences in exposure to nutrigenomically active compounds. Once exposure has occurred, the subsequent biological response depends on additional processes involving compound formation, bioavailability, metabolism, cellular signaling, and molecular targets.

### 2.4. Beyond Taste: Genetic Influences on Appetite and Eating Behavior

Although taste perception provides a particularly direct link between genotype and food choice, dietary behavior is influenced by a broader network of biological mechanisms. Genetic variability affecting appetite regulation, satiety, reward pathways, and energy balance may contribute to differences in eating-related phenotypes. Common variants at the fat mass and obesity-associated (*FTO*) locus and variants involving the melanocortin pathway, particularly melanocortin 4 receptor (*MC4R*), are among the most extensively investigated examples in relation to obesity and eating behavior [17].

However, the evidence requires careful interpretation. Associations with obesity susceptibility do not necessarily imply reproducible effects on habitual dietary intake, and studies of *FTO* and *MC4R* variants have produced heterogeneous results for food intake and preference [18,19]. More recent evidence for *MC4R* rs17782313 suggests a possible association with appetite, while evidence for increased energy intake remains less convincing [20].

These mechanisms differ conceptually from those involving sensory receptors. Whereas variants in taste-related genes can modify the perception of specific food characteristics, genetic variation affecting appetite or satiety may influence broader aspects of eating behavior without determining preference for a particular nutrient. Both mechanisms may ultimately contribute to dietary exposure, but the strength of evidence linking genotype to actual intake varies substantially among loci. Their relevance becomes particularly important in overweight and obesity, which will be considered later as a paradigmatic application of precision nutrition. Representative examples of genetic variants influencing sensory perception, food-related traits, appetite, and eating behavior, together with their potential impact on dietary exposure, are summarized in Table 1.

### 2.5. Dietary Exposure as an Interface Between Nutrigenetics and Nutrigenomics

Dietary exposure thus represents an intermediate phenotype connecting nutrigenetics and nutrigenomics. Genetic influences on sensory perception, appetite, and food preferences may contribute to differences in actual nutrient intake, within a broader environmental and sociocultural context. Once dietary components are consumed, their bioavailability, metabolism, and interactions with molecular targets introduce additional sources of variability. These successive levels of regulation provide the transition from genetically influenced dietary exposure to the nutrient-responsive molecular mechanisms discussed in the following section.

## 3. From Dietary Exposure to Nutrigenomic Response: Molecular Mechanisms

Once nutrients and bioactive food components are consumed and become biologically available, they can interact with cellular systems through multiple molecular mechanisms. Nutrients are not merely substrates for energy production or structural components of cells; they can also function as signaling molecules, precursors of regulatory metabolites or cofactors in metabolic reactions, and modulators of transcriptional and epigenetic processes. Nutrigenomics investigates these molecular responses and how they ultimately contribute to interindividual differences in metabolic and physiological phenotypes [1,2].

The transition from dietary exposure to biological response involves several interconnected steps. Following ingestion, dietary components undergo digestion, intestinal absorption, transport, biotransformation, and distribution to target tissues. At each of these levels, the concentration and chemical form of a nutrient or bioactive compound may change substantially. Consequently, dietary intake does not necessarily correspond to cellular exposure. Bioavailability and metabolism therefore represent critical interfaces between food consumption and nutrigenomic activity.

**Figure 1:**
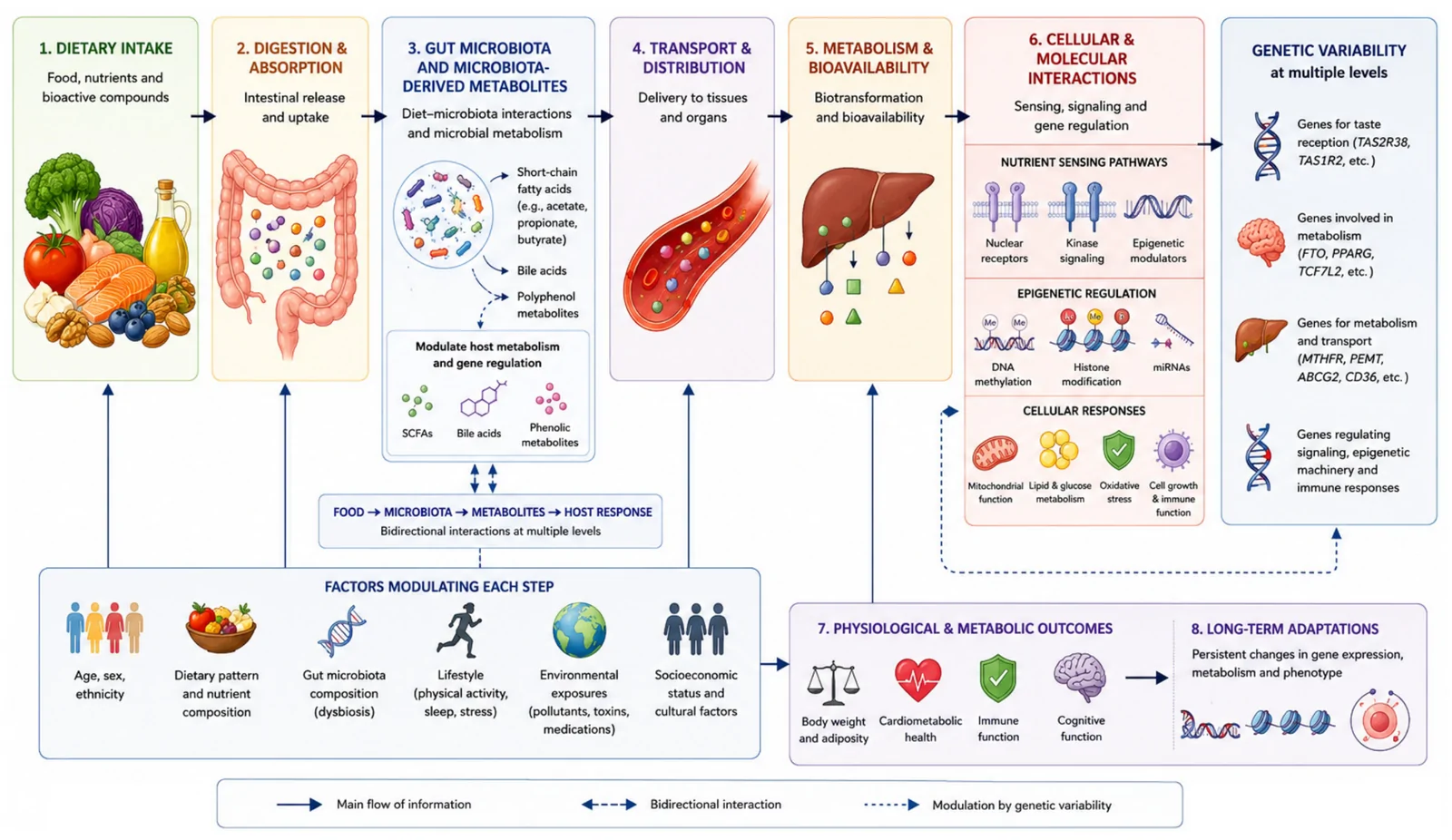
From dietary exposure to nutrigenomic response: molecular mechanisms and sources of interindividual variability. Dietary nutrients and bioactive compounds undergo sequential processes of digestion and absorption, transport and tissue distribution, and metabolic transformation that determine their effective bioavailability and cellular exposure. Once biologically available, dietary components and their metabolites can modulate nutrient- and energy-sensing pathways, transcription factors and nuclear receptors, and epigenetic mechanisms, ultimately affecting gene expression, cellular functions, and physiological and metabolic phenotypes. The gut microbiota adds an additional metabolic layer by transforming dietary substrates into bioactive metabolites, including short-chain fatty acids, secondary bile acids, and other microbial products capable of modulating host signaling, metabolism, and epigenetic regulation. Genetic variation can influence multiple levels of this pathway, from food perception and eating behavior to nutrient transport, metabolism, molecular signaling, and epigenetic regulation. These processes are further modulated by age, sex, physiological status, dietary context, lifestyle, environmental exposures, and socio-cultural factors. Overall, nutrigenomic responses emerge from interconnected biological processes in which dietary exposure is progressively modified by genetic background, microbial metabolism, and environmental and lifestyle factors, thereby contributing to interindividual variability rather than reflecting the effect of isolated nutrient–gene interactions. Arrows and connecting lines indicate the main flow of information, as specified in the figure legend.

As summarized in Figure 1, the transition from dietary exposure to nutrigenomic response can therefore be viewed as a multistep process in which digestion, absorption, transport, metabolism, molecular signaling, and gene regulation progressively connect food intake with cellular and physiological outcomes, while genetic and environmental factors can modulate each level.

### 3.1. Nutrients and Bioactive Compounds as Molecular Signals

A major mechanism underlying nutrigenomic responses is the ability of dietary components or their metabolites to modulate cellular signaling pathways and transcriptional regulators. Fatty acids, glucose, amino acids, vitamins, phytochemicals, and other food-derived molecules can influence intracellular signaling networks involved in energy sensing, oxidative stress, inflammation, lipid and glucose metabolism, and cellular homeostasis.

Some nutrients and their metabolites interact directly or indirectly with transcription factors and nuclear receptors. Fatty acids and their derivatives, for example, can modulate members of the peroxisome proliferator-activated receptor (PPAR) family, thereby affecting transcriptional programs involved in lipid metabolism, adipogenesis, inflammation, and energy homeostasis. Other nutrient-responsive regulatory systems include liver X receptors (LXRs), farnesoid X receptor (FXR), sterol regulatory element-binding proteins (SREBPs), and carbohydrate-responsive element-binding protein (ChREBP), which collectively connect changes in lipid, sterol, bile-acid, and carbohydrate availability to transcriptional control of metabolic pathways [24].

Nutrient and energy availability are also sensed through intracellular signaling systems such as AMP-activated protein kinase (AMPK) and mechanistic target of rapamycin complex 1 (mTORC1). These pathways integrate cellular energy and nutrient status with processes including glucose and lipid metabolism, protein synthesis, autophagy, and mitochondrial function [25]. Dietary and metabolic signals can additionally intersect with inflammatory pathways, including nuclear factor κB (NF-κB), thereby providing potential links between nutritional status, inflammatory signaling, and metabolic homeostasis.

Together, these mechanisms illustrate a fundamental principle of nutrigenomics: nutrients and food-derived metabolites can function as regulatory signals capable of modifying gene expression and cellular function in response to nutritional conditions.

### 3.2. Bioactive Food Compounds and Transcriptional Responses

In addition to essential nutrients, foods contain numerous bioactive compounds capable of modulating molecular pathways despite not being nutritionally essential. Polyphenols, carotenoids, isothiocyanates, and other phytochemicals have been extensively investigated for their effects on oxidative-stress responses, inflammation, xenobiotic metabolism, and gene regulation.

Sulforaphane provides a useful example linking dietary exposure to molecular response. This isothiocyanate is generated from glucoraphanin, a glucosinolate abundant in broccoli and other cruciferous vegetables, following myrosinase-dependent hydrolysis. Sulforaphane can activate cellular defense pathways, most notably the Kelch-like ECH-associated protein 1—Nuclear factor erythroid 2-related factor 2 (KEAP1–NRF2) system, resulting in NRF2-dependent transcription of genes involved in antioxidant defense, xenobiotic metabolism, and cellular stress responses [26]. Sulforaphane has also been investigated as a modulator of epigenetic mechanisms, including histone deacetylase activity [16,27].

This example connects the genetically influenced food preferences discussed in Section 2.3 with subsequent nutrigenomic responses. While variation in bitter taste perception may affect exposure to glucosinolate-containing vegetables, the biological effects of their consumption depend on isothiocyanate formation, bioavailability, metabolism, and interactions with molecular targets. These processes illustrate distinct but interconnected components of the diet–genome–phenotype pathway.

### 3.3. Metabolism and Bioavailability as Determinants of Nutrigenomic Activity

The molecular effect of a dietary component depends not only on the amount consumed but also on its digestion, absorption, biotransformation, distribution, and, where applicable, conversion into biologically active forms. Variation in intestinal transport, enzymatic activity, hepatic metabolism, tissue distribution, and elimination can therefore substantially modify effective exposure at the cellular level. Consequently, individuals consuming comparable amounts of the same food may experience different concentrations of the relevant bioactive molecules and different molecular responses.

This principle applies to both essential nutrients and food-derived bioactive compounds. Folate and other nutrients participating in one-carbon metabolism contribute to the generation of S-adenosylmethionine (SAM), the principal methyl donor used in DNA and histone methylation. Carotenoid metabolism influences the availability of retinoid-related molecules capable of regulating transcription, whereas polyunsaturated fatty acids can act as structural components, signaling molecules, and precursors of bioactive lipid mediators, whose metabolic conversion is critically influenced by delta-5 and delta-6 desaturase activities [28].

Genetic variation in enzymes and other proteins involved in these processes provides a further point of intersection between nutrigenetics and nutrigenomics. The *FADS1*/*FADS2* locus, encoding fatty acid desaturases, provides a well-characterized example: common variants in this region are strongly associated with differences in endogenous polyunsaturated fatty acid (PUFA) desaturation and consequently with circulating and tissue fatty-acid profiles [29,30]. Such findings illustrate how comparable dietary exposure may generate different internal molecular environments depending on genetic background.

This mechanistic relationship is supported by controlled dietary interventions linking *FADS1* variation to measurable metabolic responses. In the genotype-based randomized FADSDIET2 trial, 130 men homozygous for *FADS1* rs174550 were assigned to diets enriched in linoleic or α-linolenic acid for eight weeks. In the linoleic acid intervention arm, significant genotype × diet interactions were observed for the proportion of arachidonic acid in plasma phospholipids, together with selected markers of glucose–insulin homeostasis and low-grade inflammation [31]. In a separate four-week high-linoleic-acid intervention, *FADS1* rs174550 genotype was associated with baseline differences in circulating phospholipid profiles, although the observed metabolomic genotype × diet interactions did not remain significant after correction for multiple testing [32]. These findings connect genetic variation and dietary fatty acid exposure to quantifiable biochemical phenotypes, while indicating that the reproducibility and clinical significance of individual metabolic responses require further investigation.

### 3.4. Interindividual Variability in Nutrient-Responsive Pathways

Interindividual variability may also arise at the level of molecular targets and downstream signaling networks. Genetic differences affecting receptors, transcription factors, enzymes, transporters, or regulatory proteins can modify the magnitude or direction of responses elicited by dietary components. The relationship between genotype and nutrient response is therefore distributed across multiple steps of the biological pathway rather than confined to isolated gene–nutrient interactions.

Accordingly, the functional significance of a nutrigenetic variant should ideally be evaluated within the pathway connecting dietary exposure to molecular and phenotypic outcomes. Associations between individual variants and complex nutritional phenotypes are unlikely, by themselves, to provide sufficient information for precision nutrition unless they can be integrated with evidence on dietary exposure, intermediate molecular phenotypes, and measurable physiological responses.

### 3.5. From Signaling to Epigenetic Regulation

Nutrient-induced regulation of gene expression is not restricted to conventional signaling pathways and transcription factors. Cellular metabolism is directly connected to chromatin regulation because several metabolites serve as substrates or cofactors for chromatin-modifying enzymes [33]:

- One-carbon metabolism contributes to cellular SAM pools required for DNA and histone methylation;
- Acetyl-CoA provides acetyl groups for histone acetylation;
- Nicotinamide adenine dinucleotide (NAD^+^) is required for sirtuin-mediated deacetylation;
- α-ketoglutarate serves as a co-substrate for dioxygenases involved in DNA and histone demethylation.

These mechanisms provide an important interface between nutritional exposure, cellular metabolism, and regulation of gene expression. Unlike the transient activation of many signaling pathways, some epigenetic modifications can persist beyond the immediate nutritional stimulus and may contribute to longer-lasting changes in cellular state [34]. However, the stability, biological significance, and reversibility of diet-associated epigenetic changes vary considerably according to molecular context. The mechanisms through which diet and metabolism influence the epigenome are considered in the following section.

Human nutritional intervention studies illustrate both the biological relevance and the context dependence of this relationship. In a randomized, double-blind, placebo-controlled trial involving 31 patients with colorectal adenomas, supplementation with 400 μg/day of folic acid for ten weeks increased circulating folate concentrations, reduced plasma homocysteine, and increased global DNA methylation in leukocytes, whereas the corresponding change in colonic mucosa did not reach conventional statistical significance compared with placebo [35]. In contrast, a subsequent randomized intervention involving 16 healthy men with adequate dietary folate status found no substantial genome-wide methylation changes following eight weeks of folic acid supplementation, despite increased folate concentrations [36]. These observations demonstrate that nutritional modulation of one-carbon metabolism can produce measurable biochemical effects, but that changes in methylation-related metabolites do not necessarily translate into consistent epigenetic responses across tissues and populations.

## 4. Epigenetic Modulation by Diet

Epigenetic regulation represents a major molecular interface through which nutritional exposures can influence gene expression without altering the underlying DNA sequence. DNA methylation, histone modifications, chromatin remodeling, and regulatory non-coding RNAs contribute to the establishment and maintenance of transcriptional states and are sensitive, to varying degrees, to metabolic and environmental conditions. Nutrition can influence these processes both by providing substrates and cofactors required for epigenetic reactions and by modulating signaling pathways and metabolic states that regulate chromatin-associated enzymes. The epigenome therefore represents a dynamic interface connecting dietary exposure, cellular metabolism, and phenotype [37,38].

### 4.1. Nutrient Availability and DNA Methylation

DNA methylation is among the most extensively investigated mechanisms linking nutrition to epigenetic regulation. The transfer of methyl groups to cytosine residues is catalyzed by DNA methyltransferases (DNMTs) using SAM as the methyl donor. SAM availability is closely connected to one-carbon metabolism, which integrates metabolic pathways involving folate, methionine, choline, betaine, and vitamins B6 and B12 [37,39].

Consequently, nutritional status can influence the cellular environment supporting methylation reactions by altering the availability of methyl donors and related metabolites. Human observational and intervention studies support associations between nutrients involved in one-carbon metabolism and DNA methylation, although the magnitude and direction of these effects depend on nutrient, tissue, genomic region, analytical method, and study context [40]. Changes in global methylation should therefore not be considered equivalent to functionally relevant alterations at specific regulatory regions, nor should an association between nutrient status and DNA methylation necessarily be interpreted as evidence of causality.

The relationship between nutrition and DNA methylation also provides a clear example of the convergence between nutrigenomics and nutrigenetics. Methylenetetrahydrofolate reductase (*MTHFR*) represents a paradigmatic case. The common functional C677T variant (rs1801133) alters MTHFR activity and can modify the relationship between folate status and DNA methylation. Human studies have reported genotype-dependent differences in genomic DNA methylation according to folate status or controlled folate intake [41,42]. This interaction illustrates how nutrient availability and genetic background can converge on the same epigenetic pathway.

### 4.2. Diet, Cellular Metabolism, and Histone Modifications

Histone modifications provide an additional mechanism linking cellular metabolism to chromatin regulation. As outlined in Section 3.5, chromatin-modifying enzymes depend on metabolites such as acetyl-CoA, NAD^+^, and SAM, which connect cellular metabolic state with histone acetylation, deacetylation, and methylation [33,43].

This metabolic dependence provides a direct conceptual link between nutrient availability and chromatin regulation. However, changes in metabolite availability do not necessarily translate into uniform genome-wide changes in chromatin state. The effects depend on cell type, chromatin context, enzyme activity, metabolite compartmentalization, and the interaction of metabolic signals with other regulatory mechanisms.

Food-derived bioactive compounds may additionally influence chromatin-associated enzymes more directly. Sulforaphane, introduced in the preceding sections as an example linking dietary exposure with molecular response, has been investigated for its ability to modulate histone deacetylase activity [27]. Polyphenols and other dietary bioactives have likewise been associated with modulation of chromatin-related enzymes and signaling pathways. However, experimental concentrations, human bioavailability, tissue exposure, and the extent to which these molecular effects translate into measurable physiological outcomes remain essential considerations when interpreting such findings. Additional experimental evidence supports the potential epigenetic effects of other dietary bioactive compounds. For example, pterostilbene, a naturally occurring stilbenoid present in foods such as blueberries and grapes, was associated with genome-wide changes in DNA methylation in a human neuroblastoma cell model [44].

### 4.3. Nutritional Regulation of Non-Coding RNAs

Dietary exposures may also influence gene regulation through non-coding RNAs. MicroRNAs (miRNAs), in particular, regulate gene expression post-transcriptionally by modulating mRNA stability and translation and participate in metabolic pathways involved in lipid homeostasis, glucose metabolism, inflammation, adipogenesis, and cellular stress responses [45,46].

Human studies indicate that circulating and tissue miRNA profiles can change in response to meal challenges and sustained dietary interventions. However, substantial heterogeneity in dietary interventions, study populations, biological samples, analytical methods, and candidate miRNAs currently limits comparability across studies and the identification of reproducible diet-responsive signatures [47].

Long non-coding RNAs and other regulatory RNA species may also participate in diet-responsive molecular pathways, although their functional relevance in human precision nutrition remains less clearly established. More generally, non-coding RNAs are of interest as potential biomarkers of nutritional exposure or response, but their use for this purpose requires reproducibility, biological validation, and methodological standardization.

### 4.4. Developmental Programming and Persistence of Diet-Induced Epigenetic Changes

The effects of nutritional exposure on the epigenome may be particularly relevant during developmental periods characterized by extensive epigenetic remodeling. Prenatal development and early postnatal life represent critical windows during which environmental and nutritional signals may produce molecular effects with long-term consequences, providing an important mechanistic dimension to the developmental origins of health and disease.

A landmark human example is provided by individuals exposed prenatally to the Dutch Hunger Winter of 1944–1945. Periconceptional exposure to famine was associated, approximately six decades later, with lower DNA methylation at the imprinted Insulin-like growth factor 2 (*IGF2*) locus compared with unexposed same-sex siblings [34]. This observation provides evidence that nutritional conditions during a critical developmental window can be associated with persistent epigenetic differences in humans, although it does not by itself establish that the observed methylation difference mediates subsequent disease phenotypes.

Such findings support the biological plausibility of nutritional programming but also illustrate the difficulty of establishing causal chains from nutritional exposure through a specific epigenetic modification to a later phenotype. Genetic background, developmental timing, lifestyle, socioeconomic conditions, and other environmental exposures can all contribute to the observed relationships.

An important characteristic of epigenetic regulation is its dynamic nature. In contrast to germline DNA sequence variation, many epigenetic marks can change over time and in response to physiological or environmental conditions. This plasticity has generated considerable interest in the possibility that dietary interventions might modify unfavorable epigenetic states. However, plasticity should not be equated with simple reversibility: the stability and responsiveness of epigenetic marks vary according to cell type, genomic locus, developmental stage, and biological context. Evidence that diet is associated with an epigenetic marker therefore does not imply that dietary modification will necessarily reverse either the marker or the associated phenotype.

Representative examples illustrating how nutritional exposures can interact with epigenetic mechanisms, together with the corresponding molecular links, available evidence, and major interpretative limitations, are summarized in Table 2.

### 4.5. Epigenetic Variability in the Framework of Precision Nutrition

Epigenetic information adds an important dimension to precision nutrition because it may capture biological consequences of interactions among genotype, diet, environment, and metabolic state. Whereas germline genetic variation is essentially stable throughout life, epigenetic profiles are dynamic, tissue- and cell-type-dependent, and may reflect both previous and current exposures [38].

This distinction suggests complementary roles for genetic and epigenetic information. Genetic variants may help identify relatively stable differences in predisposition or nutrient processing, whereas epigenetic profiles may provide information on biological state or response to nutritional and environmental exposures. Integration of these layers with transcriptomic, metabolomic, microbiome, and phenotypic data could therefore provide a more informative representation of individual nutritional responses than genetic information alone.

At the same time, epigenetic biomarkers present substantial methodological challenges. DNA methylation and other epigenetic marks are cell-type- and tissue-specific, vary with age and physiological state, and may represent causes, consequences, or correlates of metabolic phenotypes. Measurements obtained from peripheral blood, although readily accessible, may not reflect regulatory changes occurring in metabolically relevant tissues such as liver, adipose tissue, skeletal muscle, or intestine. Longitudinal designs, repeated measurements, appropriate tissue selection, and independent validation will therefore be necessary before epigenetic signatures can be reliably incorporated into predictive models for precision nutrition [38].

Overall, diet-sensitive epigenetic mechanisms provide a biologically plausible link between nutritional exposure and changes in gene regulation. Their integration with genetic variability reinforces the concept that individual responses to diet arise from interactions between relatively stable genomic determinants and dynamic molecular states. This multi-layered relationship becomes even more complex when the gut microbiota is considered, as microbial metabolism can modify the availability of nutrients and bioactive metabolites capable of influencing both host signaling pathways and epigenetic regulation.

## 5. The Gut Microbiota as an Additional Layer of Diet–Genome Interaction

The gut microbiota represents an additional source of interindividual variability in the relationship between dietary exposure and host molecular response. Dietary components reaching the intestine interact with a highly complex microbial ecosystem whose composition and functional capacity are influenced by diet, medication, lifestyle, age, host-related factors, and numerous environmental exposures. Diet itself is a major determinant of gut microbiome composition and function [49]. In turn, microbial metabolism can modify the chemical form and bioavailability of dietary compounds and generate metabolites capable of interacting with host metabolic, signaling, and epigenetic pathways. The microbiota can therefore be viewed as a dynamic metabolic interface linking dietary exposure to host physiology.

### 5.1. Microbial Metabolism as a Modifier of Dietary Exposure

The biological exposure of host tissues to dietary components is not determined exclusively by intestinal absorption of the molecules originally present in food. Gut microorganisms can metabolize otherwise poorly digestible substrates and transform dietary compounds into molecules with different biological activities. Fermentation of dietary fiber, for example, generates short-chain fatty acids (SCFAs), principally acetate, propionate, and butyrate, which can act as energy substrates and signaling molecules [50]. Microbial metabolism also contributes to the transformation of bile acids, polyphenols, amino acids, and other dietary constituents, thereby expanding the range of metabolites potentially available to interact with host cells [7].

Differences in microbiota composition and metabolic capacity may generate distinct metabolite profiles even among individuals consuming comparable diets. Biologically relevant exposure therefore includes not only ingested dietary compounds but also the host- and microbiota-derived metabolites produced during their processing.

### 5.2. Microbiota-Derived Metabolites and Host Molecular Responses

Once generated, microbial metabolites can influence host physiology through multiple mechanisms. SCFAs provide a particularly illustrative example because they act not only as metabolic substrates but also as signaling molecules through G protein-coupled receptors, influencing intestinal barrier function, immune regulation, and host metabolism. Butyrate can additionally inhibit histone deacetylase activity, providing a direct mechanistic link between microbial metabolism and the epigenetic regulation discussed in the preceding section [50,51].

Experimental and clinical evidence provides a direct illustration of this connection between microbial metabolites and host physiology. Chambers et al. demonstrated that propionate stimulated peptide YY (PYY) and glucagon-like peptide-1 (GLP-1) secretion in primary human colonic cell cultures. In an acute randomized crossover intervention, targeted colonic delivery of propionate through an inulin–propionate ester increased postprandial circulating PYY and GLP-1 concentrations and reduced energy intake. Furthermore, a 24-week randomized intervention involving 60 overweight adults reported reduced weight gain and favorable changes in selected measures of adiposity and metabolic regulation compared with an inulin control [52]. These findings provide a mechanistic and physiological link between a microbiota-derived metabolite, enteroendocrine signaling, and measurable metabolic outcomes. However, targeted colonic propionate delivery should not be regarded as equivalent to conventional dietary fiber supplementation, and the generalizability of these results requires further investigation.

Other microbiota-derived or microbiota-modified molecules, including secondary bile acids and metabolites generated from amino acids and dietary phytochemicals, can interact with host receptors, metabolic pathways, and transcriptional regulatory systems. Microbial metabolism therefore constitutes an intermediate layer between dietary exposure and host molecular responses, converting or modifying dietary substrates into signals capable of influencing host regulatory pathways [7].

These interactions are bidirectional: diet shapes microbial composition and function, while microbial metabolism modifies the bioactive compounds available to the host. The resulting molecular responses therefore reflect a dynamic diet–microbiota–host relationship rather than a simple linear pathway.

### 5.3. The Microbiota in Precision Nutrition

The substantial interindividual variability in the gut microbiota has stimulated considerable interest in its potential contribution to predicting individual responses to dietary interventions. An influential example was provided by Zeevi et al. [53], who integrated clinical, dietary, behavioral, and microbiome features to predict individualized postprandial glycemic responses and demonstrated substantial interindividual variability in glycemic responses to identical meals [53].

The subsequent PREDICT 1 study extended this concept to postprandial glucose, insulin, and triglyceride responses in a large cohort of twins and unrelated individuals. Person-specific factors, including gut microbiome characteristics, contributed to the prediction of postprandial responses, although their relative importance differed according to the metabolic outcome considered. Notably, genetic variation contributed modestly to several of these predictive models, emphasizing that no single biological layer adequately captures interindividual variability in dietary response [54].

However, the translation of microbiome associations into reliable predictive tools remains challenging. Microbiota composition is dynamic and strongly influenced by environmental and lifestyle factors, while results can also depend on sampling procedures, sequencing approaches, bioinformatic pipelines, and whether taxonomic or functional features are considered. Moreover, associations between microbial profiles and metabolic phenotypes do not necessarily establish causality, and taxonomic composition alone may provide an incomplete representation of microbial metabolic activity [7].

For these reasons, the microbiota should not be considered an isolated predictor of dietary response but rather an additional layer within the broader diet–genome–phenotype framework. Its potential contribution to precision nutrition is likely to emerge from integration with dietary exposure, host genetic variation, epigenetic and transcriptomic profiles, metabolomic measurements, and well-characterized phenotypic outcomes. Host germline variation represents a relatively stable component of interindividual variability, whereas the gut microbiota is considerably more dynamic and environmentally responsive; indeed, environmental factors may exert a stronger influence on microbiome composition than host genetics in many human populations [49].

## 6. Integrating Nutrigenetics and Nutrigenomics: The Diet–Genome–Phenotype Model

The preceding sections illustrate how dietary exposure and its biological consequences are influenced by genetic, behavioral, environmental, metabolic, epigenetic, and microbial factors. Their integration requires a framework that accounts for variability both in actual dietary exposure and in the molecular and physiological responses to that exposure [5].

We propose that nutrigenetics and nutrigenomics can be conceptually integrated within a diet–genome–phenotype model, in which genetic variation can act both upstream and downstream of dietary exposure. Upstream, genetic differences may influence sensory perception, food preferences, appetite, and dietary behavior, thereby contributing to determining which nutrients and bioactive compounds are actually consumed. Downstream, genetic variation can modify their absorption, transport, metabolism, molecular targets, and cellular responses. At and across these levels, the epigenome, gut microbiota, transcriptome, and metabolome represent dynamic molecular layers through which dietary and environmental exposures can further shape biological responses. This multilayer perspective is increasingly central to precision nutrition research, in which genomic information is considered together with molecular, microbial, behavioral, and environmental data rather than as an isolated determinant of dietary response [5].

### 6.1. From Genotype to Dietary Exposure and from Dietary Exposure to Phenotype

Within this framework, two complementary dimensions of gene–diet interaction can be distinguished. The first concerns the influence of genetic variation on dietary exposure. Variants affecting taste perception, food preference, appetite, or eating behavior can modify the probability, quantity, and frequency of consumption of specific foods. Genetic variability may therefore contribute not only to differences in biological response but, in some cases, also to differences in the exposure itself.

The second dimension concerns the influence of genetic variation on the biological response to dietary exposure. Once nutrients or bioactive compounds have been consumed, differences in digestion, absorption, transport, enzymatic conversion, signaling, transcriptional regulation, and metabolic pathways may generate heterogeneous molecular and physiological responses among individuals exposed to comparable diets.

These two dimensions can be summarized as an interconnected sequence:

genetic background → food perception and behavior → dietary exposure → bioavailability and metabolism → molecular and epigenetic response → metabolic phenotype.

However, this representation is necessarily simplified. Feedback mechanisms operate throughout the system: metabolic and physiological states can influence appetite and food preferences; dietary patterns can alter gut microbiota composition and function as well as epigenetic and metabolic profiles; and the resulting molecular changes may subsequently influence responses to future dietary exposures. The diet–genome–phenotype relationship should therefore be viewed as a dynamic and multidirectional network rather than as a unidirectional pathway.

Within this model, nutrigenetics and nutrigenomics describe complementary and interconnected processes. Genetic variation may influence both dietary exposure and the biological response to that exposure, while nutrient-responsive molecular changes can modify the conditions under which subsequent dietary exposures are processed.

The distinctive contribution of the proposed framework is therefore not the identification of previously unrecognized diet–genome interactions, but the explicit distinction and integration of genetic determinants of dietary exposure and genetic determinants of the biological response to that exposure. This distinction provides a basis for testable hypotheses: variants influencing food perception or appetite may primarily affect actual nutrient intake, whereas variants involved in nutrient metabolism or signaling may modify molecular and metabolic responses under controlled dietary exposure. Future studies could examine these pathways separately and jointly, combining quantitative dietary assessment, genotype-stratified interventions, and longitudinal molecular and phenotypic measurements. Such investigations would help determine whether genetic effects operate predominantly through dietary behavior, biological response, or both.

### 6.2. Stable and Dynamic Components of Individual Nutritional Response

An important feature of this model is the coexistence of biological determinants characterized by markedly different degrees of temporal stability. Germline genetic variation represents a relatively stable component of interindividual variability. By contrast, dietary intake, physiological and metabolic status, epigenetic profiles, transcriptomic and metabolomic states, and gut microbiota composition are dynamic and can change over time in response to age, lifestyle, physiological conditions, environmental exposures, medications, disease, and dietary interventions.

Germline genotype may provide information on relatively stable predispositions, whereas dynamic molecular, metabolic, and microbial measurements can reflect current or previous exposures and physiological states. Integrating these complementary sources of information may improve the characterization of individual nutritional responses, although tissue specificity, temporal variability, methodological heterogeneity, and reproducibility remain important challenges. Multi-omics approaches offer opportunities for such integration, but their predictive contribution requires independent validation and demonstration of added value beyond simpler clinical and dietary models [5,55].

### 6.3. From Population-Based Nutrition to Precision Nutrition

Population-based nutritional recommendations remain the foundation of public-health nutrition strategies and are supported by evidence derived from large populations. Precision nutrition should therefore not be viewed as an alternative to population-based recommendations, but as a complementary approach aimed at characterizing and, where sufficiently supported by evidence, predicting meaningful interindividual variability in responses to dietary exposures and interventions [5].

The central challenge is to determine which components of this variability are reproducible, biologically meaningful, and sufficiently predictive to inform individualized interventions. A statistically significant association between a genetic variant and a nutritional phenotype does not necessarily imply that the variant has useful predictive value, nor that genotype-based dietary modification will improve behavioral, metabolic, or clinical outcomes. Indeed, evidence supporting the incorporation of genetic information into nutritional interventions remains limited, with substantial heterogeneity among studies in the variants examined, nutritional advice provided, and outcomes measured [56].

The Food4Me randomized controlled trial provides an informative example of both the potential and the limitations of personalization. Personalized nutritional advice produced greater improvements in several dietary behaviors than conventional population-based advice [57]. However, increasing the level of personalization by incorporating phenotypic and genotypic information did not consistently produce additional benefits, and genotype-based personalization did not result in greater weight loss than personalized advice without genetic information [57].

The Food4Me findings underscore the distinction between biological personalization and demonstrated clinical utility. Incorporating genetic or molecular information into nutritional recommendations is justified only when its additional contribution is supported by reproducible evidence, prospective validation, and measurable improvements in prediction or intervention outcomes.

From this perspective, the diet–genome–phenotype model proposed here should be regarded primarily as a conceptual and research framework rather than an immediately actionable clinical algorithm. Its purpose is to identify the major biological levels that may contribute to interindividual variability in dietary response and to provide a structure for integrating genomic information with dietary exposure, dynamic molecular biomarkers, the gut microbiota, environmental factors, and phenotypic measurements.

Complex metabolic conditions provide particularly informative settings in which to examine this framework. Overweight and obesity arise from interactions among genetic susceptibility, dietary behavior, energy balance, metabolic regulation, environmental exposures, and lifestyle. They therefore represent a useful paradigmatic model for evaluating whether selected genetic information, integrated with dietary and molecular phenotypes, can contribute to the development and validation of precision nutrition strategies.

## 7. Overweight and Obesity as a Paradigmatic Application of Precision Nutrition

Overweight and obesity provide a useful setting for examining the potential and limitations of precision nutrition. Their multifactorial etiology involves genetic susceptibility, dietary behavior, neuroendocrine and metabolic regulation, physical activity, and environmental and socioeconomic factors. These interacting determinants contribute to differences in both susceptibility to weight gain and responses to nutritional interventions [58].

The genetic contribution to obesity encompasses a broad spectrum, ranging from rare, highly penetrant variants causing monogenic forms of obesity to the cumulative effects of numerous common variants individually associated with modest differences in body weight and related metabolic traits [58]. The latter are particularly relevant to common overweight and obesity but, considered individually, generally have limited predictive value. For precision nutrition, therefore, the relevant question is not simply whether a variant is associated with obesity, but whether it identifies a reproducible biological mechanism related to dietary exposure or modifies the response to a defined nutritional intervention.

### 7.1. An Evolutionary Perspective on Genetic Susceptibility to Obesity

From an evolutionary perspective, genetic susceptibility to obesity and related metabolic phenotypes can be interpreted as the outcome of genetic variation acting within changing environmental and nutritional contexts. Genetic variants currently associated with obesity susceptibility should not be regarded as having evolved specifically to promote excessive adiposity; rather, they affect physiological systems whose fitness consequences have varied according to environmental and nutritional conditions. The classical *thrifty genotype hypothesis* proposed that genetic variants favoring efficient energy utilization and fat storage could have conferred a selective advantage during recurrent periods of food scarcity, while becoming detrimental in modern environments characterized by sustained food availability and reduced energy expenditure [59]. This interpretation has subsequently been questioned, particularly regarding whether famine-related selective pressures were sufficiently strong and recurrent to account for the present distribution of obesity-predisposing alleles. The alternative *drifty gene hypothesis* proposes that the relaxation of selective constraints on the upper limits of adiposity may instead have allowed genetic variation predisposing to obesity to accumulate largely through genetic drift [60].

More recent evolutionary perspectives emphasize that these hypotheses need not provide mutually exclusive explanations and focus greater attention on evolutionary mismatch, whereby genetic and physiological adaptations shaped under ancestral nutritional and ecological conditions interact with rapidly changing modern environments [61]. In this context, present-day obesity susceptibility can be viewed as an emergent property of interactions between inherited biological variation and environmental conditions rather than as the consequence of specific “obesity genes”. More broadly, the genomic architecture underlying complex phenotypes ultimately reflects evolutionary processes that generate and shape genetic variation, including mutation, gene duplication and functional diversification, natural selection, and genetic drift [62].

These evolutionary perspectives emphasize the context-dependent expression of genetic susceptibility. Variants associated with energy storage, appetite, food preferences, or nutrient metabolism may have different phenotypic consequences under different nutritional and environmental conditions. This context dependence is particularly relevant when interpreting genetic associations in precision nutrition research.

### 7.2. Genetic Determinants of Dietary Behavior and Energy Intake

As discussed in Section 2, variation in *TAS2R38*, *TAS1R2*/*TAS1R3*, and *CD36* has been associated with differences in bitter, sweet, and lipid-related taste perception. Although effects on habitual food consumption are less consistently demonstrated, these loci illustrate how sensory genetic variation may contribute to dietary exposure.

In obesity, however, dietary exposure is influenced not only by sensory perception but also by central mechanisms regulating hunger, satiety, reward, and energy intake. Common variation at the *FTO* locus represents one of the most extensively investigated examples of genetic susceptibility to common obesity. In the POUNDS Lost trial, variation at *FTO* was associated with differential changes in appetite according to dietary protein intake, illustrating the possibility that genotype may interact with dietary composition at the level of eating-related phenotypes [23].

The melanocortin pathway provides another biologically compelling example. Rare functional variants in *MC4R* can cause severe monogenic obesity, whereas common variation near *MC4R*, particularly rs17782313, is associated with common obesity susceptibility [63]. Studies have investigated associations between this variant and eating-related phenotypes, although evidence for its effect on actual energy intake remains less consistent. A recent systematic review and meta-analysis found suggestive evidence of increased appetite among carriers of the C risk allele, although the association was significant only under a fixed-effects model, whereas no significant association with energy intake was observed under the random-effects model [20]. These findings emphasize the distinction between an intermediate behavioral phenotype, such as appetite, and actual dietary exposure.

These examples distinguish genetic influences on food-specific sensory perception from those affecting appetite, satiety, and energy intake. Both may contribute to dietary exposure through different mechanisms. Their relevance to precision nutrition should therefore be evaluated in relation to the specific phenotype under investigation, without assuming that an association with dietary behavior necessarily predicts a genotype-dependent response to nutritional intervention.

### 7.3. Genetic Variability in Metabolic Response to Diet

A second level of variability emerges after dietary exposure has occurred. Individuals consuming comparable amounts and types of nutrients may differ in their metabolic responses because of genetic variation affecting nutrient absorption, transport, enzymatic conversion, signaling pathways, or metabolic regulation.

Lipid metabolism provides several useful examples. Apolipoprotein E (*APOE*) genotype influences circulating lipid profiles and has been extensively investigated as a potential modifier of responses to dietary fat. Controlled dietary studies have reported genotype-dependent differences in selected lipid and inflammatory responses, although diet itself may exert a larger effect than genotype on several outcomes [64,65]. These findings illustrate both the biological plausibility and the limitations of translating an established metabolic genotype into personalized dietary recommendations.

The *FADS1*/*FADS2* locus provides a particularly informative example because genetic variation directly affects endogenous PUFA metabolism [29,30]. Importantly, genotype-dependent responses have also been demonstrated experimentally. In the genotype-based randomized FADSDIET2 trial, *FADS1* rs174550 modified metabolic responses to diets enriched in linoleic or α-linolenic acid, providing direct evidence that variation in fatty-acid metabolism can influence response to defined dietary exposures [31].

Variation in peroxisome proliferator-activated receptor gamma (*PPARG*), a central regulator of adipocyte differentiation and lipid metabolism, has likewise been investigated in relation to dietary fat. Interactions involving the Pro12Ala polymorphism and dietary fatty-acid composition have been reported in relation to lipid and metabolic phenotypes [66], although the consistency and predictive relevance of such interactions require further evaluation.

Transcription factor 7-like 2 (*TCF7L2*), one of the loci most strongly associated with susceptibility to type 2 diabetes, provides an informative example of variant-specific evidence for gene–diet interactions. For rs7903146, genotype-dependent responses have been reported in randomized dietary interventions, including PREDIMED, and a pooled individual-participant analysis of seven randomized trials identified a significant interaction between diet/lifestyle intervention and genotype for changes in fasting glucose [67,68]. Evidence for rs12255372 is more limited but includes the POUNDS Lost trial, in which the variant interacted with dietary fat intake in relation to changes in body composition and glycemic outcomes at 6 months, although these interactions were not maintained at 24 months [69]. These findings illustrate how the strength of nutrigenetic evidence may differ between variants within the same locus and support variant-specific rather than gene-level evaluation.

Together, these examples illustrate the need to distinguish metabolic associations from experimentally supported genotype–diet interactions. Their potential predictive relevance must be evaluated at the variant level, considering the reproducibility, magnitude, and consistency of the observed effects.

### 7.4. From Genetic Susceptibility to Differential Response to Weight-Loss Interventions

The potential clinical relevance of nutrigenetics in obesity ultimately depends on whether genetic information improves prediction of individual responses to intervention. Weight-loss responses to apparently comparable dietary programs show substantial interindividual variability, including differences in the magnitude of weight reduction, body composition, glycemic control, lipid profiles, and long-term weight maintenance.

Part of this variability may reflect genetic differences, but isolating genotype-dependent effects is difficult. Dietary adherence, baseline metabolic status, physical activity, medication, age, sex, microbiota composition, socioeconomic factors, and behavioral characteristics can all influence outcomes. Furthermore, many reported gene–diet interactions derive from observational analyses or secondary analyses of intervention studies and have not been consistently replicated.

The DIETFITS randomized clinical trial provides an instructive example. Despite substantial interindividual variability in weight loss, neither a predefined three-SNP genotype pattern nor baseline insulin secretion identified individuals who achieved greater weight loss with a healthy low-fat versus a healthy low-carbohydrate diet [70]. This negative result is particularly relevant because it demonstrates that biological plausibility and retrospective associations are insufficient to establish the utility of genotype-guided dietary assignment.

Conversely, individual genotype–diet interactions have been reported in controlled interventions. As noted above, *FTO*, *TCF7L2*, and *FADS1* provide examples in which genotype has been associated with differential behavioral or metabolic responses to specific dietary conditions. Such findings are valuable for hypothesis generation, but replication, effect-size estimation, and demonstration of incremental predictive value remain necessary before they can support individualized dietary recommendations.

For precision nutrition, the distinction between genetic susceptibility and differential response to intervention is essential. Variants associated with obesity risk may have limited value for predicting dietary responses, whereas reproducible genotype–diet interactions could provide more directly relevant information. Their usefulness nevertheless depends on demonstrated incremental predictive value beyond established clinical and behavioral variables.

The same reasoning applies to polygenic susceptibility. Polygenic scores aggregate information across many common variants and can improve the estimation of genetic predisposition to obesity, but prediction of susceptibility and prediction of treatment response are distinct objectives. Recent prospective genotype-stratified intervention studies have begun to test directly whether genome-wide polygenic susceptibility modifies the weight-loss response to dietary intervention. In a recent genetic-first randomized controlled trial, individuals from the extremes of a BMI polygenic-score distribution were prospectively recruited to evaluate whether genetic predisposition influences the BMI response to a dietary intervention [71]. Such approaches underscore the importance of prospective rather than retrospective evaluation before polygenic information can be incorporated into precision nutrition strategies.

### 7.5. Toward a Research-Oriented Genetic Panel for Overweight and Obesity

The considerations above provide a rationale for exploring whether a selected set of genetic variants could be developed into a standardized research tool for investigating precision nutrition in overweight and obesity. Such a panel should be conceptually and operationally distinct from diagnostic panels designed to identify monogenic obesity and should not be regarded as a clinically validated nutrigenetic test. Targeted next-generation sequencing (NGS) panels provide an established technical strategy for the simultaneous analysis of predefined sets of genes and variants and offer advantages in terms of sequencing depth, analytical focus, and streamlined data interpretation compared with broader genomic approaches [72].

Rather than including a large number of obesity-associated loci solely on the basis of genome-wide association signals, variant selection should be driven by evidence connecting genotype to specific components of the diet–genome–phenotype pathway. Candidate variants could therefore represent distinct functional domains, including sensory perception and food preference, appetite and satiety, nutrient absorption and metabolism, lipid and glucose homeostasis, bioactive-compound metabolism, and nutrient-responsive molecular pathways.

The proposed selection is illustrative rather than exhaustive and is intended to capture complementary biological pathways and different levels of nutrigenetic evidence within a manageable research-oriented panel. Candidate variants were selected on the basis of their documented functional or phenotypic relevance, the availability of human studies addressing nutritional or metabolic outcomes, and their potential to illustrate the distinction between genetic susceptibility and genotype-dependent dietary responses. Other obesity- or nutrition-associated loci may be equally or more strongly supported for particular outcomes and should be considered in future extensions or reassessments of the panel.

A research-oriented panel could consequently include selected candidate variants in genes such as *TAS2R38*, *TAS1R2*, *CD36*, *FTO*, *MC4R*, *LEP*, *APOE*, *FADS1*, *PPARG*, and *TCF7L2*, representing complementary components of the diet–genome–phenotype pathway. Importantly, inclusion in such a panel would not imply equivalent levels of evidence, predictive value, or clinical relevance. Representative candidate variants spanning these functional domains, together with the principal evidence supporting their potential inclusion in a research-oriented panel, are summarized in Table 3.

Observational studies of *LEP* rs7799039 further illustrate the importance of evaluating genetic variation within a broader phenotypic and nutritional context. In young healthy adults, this promoter variant has been investigated in relation to body-composition measures, including total body mineral estimates and fat mass, alongside nutritional and anthropometric variables. The reported association with body mineral estimates, but not fat mass, highlights the phenotype-specific nature of genetic associations and the importance of considering relevant covariates [81]. Such findings should not be interpreted as evidence of a genotype-dependent response to dietary intervention, but rather as support for integrating genetic information with nutritional, anthropometric, and metabolic assessments.

The value of such a panel would derive primarily from its integration with carefully characterized phenotypic information. Genotype should therefore be evaluated together with quantitative dietary assessment, anthropometric measurements, metabolic biomarkers, lifestyle variables, and, where appropriate, transcriptomic, epigenetic, metabolomic, or microbiome data. Longitudinal and intervention studies could then determine whether individual variants, combinations of variants, or integrated multilayer models predict response to specific dietary interventions.

The proposed panel should remain iterative, allowing candidate variants to be added, reassessed, or removed as new evidence becomes available. Rather than constituting a finalized clinical test, it provides a research framework for prospective evaluation of genotype–diet interactions and for determining whether selected genetic markers contribute incremental predictive information beyond conventional clinical, dietary, behavioral, and metabolic variables.

### 7.6. Obesity as a Test Case for the Transition Toward Precision Nutrition

Overweight and obesity provide a practical setting for investigating the transition from nutrigenetic associations to validated precision nutrition strategies. Their measurable phenotypes, modifiable dietary exposures, and well-characterized metabolic pathways offer opportunities to test candidate genotype–diet interactions prospectively. However, translating these findings into individualized recommendations requires evidence of reproducibility, incremental predictive value, and improved outcomes compared with conventional nutritional approaches. These requirements, together with the methodological and clinical limitations of current evidence, are examined in the following section.

## 8. From Association to Actionability: Current Evidence and Limitations

The increasing number of reported associations between genetic variants, dietary exposures, and metabolic phenotypes has raised considerable expectations regarding the development of genetically informed nutritional recommendations. However, the transition from identifying a statistically significant gene–diet interaction to using genetic information to guide an individualized dietary intervention requires several additional levels of evidence. Association, biological plausibility, predictive performance, clinical utility, and actionability represent distinct concepts and should not be considered interchangeable [82,83].

This challenge is particularly relevant to complex traits such as obesity, where modest genetic effects and substantial environmental and metabolic heterogeneity can limit the predictive value of individual variants, even when their associations are reproducible.

### 8.1. From Statistical Association to Biological Relevance

A statistically significant association between a genetic variant and a nutritional or metabolic phenotype represents an initial level of evidence but does not necessarily establish a functional relationship. Associations may reflect linkage disequilibrium with the causal variant, population structure, residual confounding, or other sources of bias and may also arise from inadequate control for multiple testing. Moreover, statistically robust effects may be too small to have meaningful predictive value at the individual level.

Functional characterization is therefore important for establishing biological plausibility. Ideally, a candidate variant should be linked to a defined molecular mechanism involving gene expression, protein function, nutrient metabolism, sensory perception, signalling, or another component of the diet–genome–phenotype pathway. Evidence from molecular studies can substantially strengthen the interpretation of epidemiological associations, although biological plausibility alone does not demonstrate that a variant is useful for nutritional stratification [82].

Evidence of a genotype–diet interaction requires demonstrating that the association between dietary exposure and a defined outcome differs according to genotype. Such findings must be independently replicated before their predictive relevance can be established.

An important distinction should also be maintained between statistical association, evidence of a causal biological mechanism, and evidence of a causal effect of dietary intervention. Observational genotype–phenotype and genotype–diet associations may generate biologically plausible hypotheses but cannot, by themselves, establish causal relationships. Functional experiments can support specific molecular mechanisms, whereas appropriately designed randomized dietary interventions provide stronger evidence for the effects of nutritional exposure and for genotype-dependent differences in those effects. Nevertheless, even a replicated genotype × diet interaction does not establish that genotype-guided dietary recommendations improve clinical outcomes. Such conclusions require prospective studies directly evaluating the benefit of genotype-informed interventions against appropriate non-genetic alternatives.

### 8.2. Replication and Heterogeneity of Gene–Diet Interactions

Replication remains a major challenge in nutrigenetic research. Gene–diet interactions may depend on population ancestry, age, sex, baseline nutritional status, dietary patterns, environmental exposures, and other characteristics of the study population. Differences in dietary assessment methods, definitions of nutritional exposure, phenotype measurement, statistical models, and sample size can further contribute to heterogeneous results.

Interaction analyses generally require greater statistical power than analyses of main genetic effects, and many nutrigenetic studies remain relatively small. Limited statistical power can result in a failure to detect genuine interactions and in unstable effect estimates, whereas multiple testing and selective reporting may increase the likelihood that apparently significant genotype-dependent effects fail to replicate [84].

Replication should therefore involve not simply the observation of an association with the same variant, but also consistency in the direction of the effect, dietary exposure, phenotype, and overall pattern of the interaction. Evidence obtained across independent populations and, where possible, through systematic review, meta-analysis, or well-powered consortia is particularly important before a gene–diet interaction can be considered sufficiently robust for predictive applications.

Recent analyses of polygenic risk–diet interactions in obesity illustrate this problem. Although individual observational studies have reported interactions between obesity-related polygenic scores and dietary factors, pooled evidence remains heterogeneous, and meta-analyses have not consistently confirmed interactions for several commonly studied nutrient exposures [85].

Population diversity represents an additional challenge. Allele frequencies, linkage disequilibrium patterns, dietary exposures, and environmental contexts differ among populations, potentially limiting transferability. Genetic models developed predominantly in populations of European ancestry may perform differently in other groups, making ancestral, geographic, and population diversity essential for the development of broadly applicable precision nutrition models [85,86].

### 8.3. High-Dimensional Modeling and Uncertainty in Gene–Diet Interaction Studies

The identification of genotype–diet interactions presents substantial statistical challenges, particularly when large numbers of genetic variants, dietary exposures, and potential interaction terms are evaluated simultaneously. Conventional hypothesis-testing approaches may have limited power in this setting, while multiple comparisons, correlations among predictors, and small interaction effects increase the risk of false-positive findings and unstable estimates. Moreover, the number of candidate interaction terms can greatly exceed the available sample size, making variable selection and model regularization important considerations.

High-dimensional approaches, including penalized regression and structured variable-selection methods, offer alternatives to testing individual interactions separately [87]. These methods can jointly evaluate genetic main effects, dietary factors, and their interactions while reducing model complexity and potentially improving the identification of relevant predictors. Hierarchical selection strategies may also be useful for preserving relationships between interaction terms and their corresponding main effects. Nevertheless, the selection of an interaction term does not establish its biological relevance, causal interpretation, or predictive utility. Model stability, appropriate adjustment for confounding, independent replication, and external validation remain essential.

An additional challenge concerns the uncertainty associated with estimated interaction effects. Confidence intervals, resampling procedures, and sensitivity analyses can help characterize the precision and robustness of conventional estimates. Bayesian variable-selection approaches provide a complementary framework by incorporating prior information and expressing uncertainty through posterior distributions and inclusion probabilities [88]. Such methods may be particularly useful when multiple correlated predictors are considered or when interaction estimates are unstable. However, posterior inclusion probabilities depend on model specification and prior assumptions and should not be interpreted as probabilities of biological causality.

Within the proposed diet–genome–phenotype framework, these statistical approaches could support the identification and prioritization of candidate genotype–diet interactions for subsequent experimental investigation. Importantly, uncertainty should be considered not only during initial interaction discovery but also when evaluating potential predictive applications. When estimated effects are small or close to thresholds used for research prioritization or clinical decision-making, uncertainty intervals and sensitivity to modeling assumptions should be explicitly reported. Such analyses would help distinguish statistically selected interactions from reproducible effects with sufficient precision and incremental predictive value to justify further investigation.

### 8.4. Observational Evidence Versus Nutritional Intervention

Observational studies are essential for identifying candidate gene–diet interactions but have intrinsic limitations in establishing whether genetic information can guide dietary intervention. Dietary intake is difficult to measure accurately, and associations may be affected by residual confounding, reverse causality, correlations among dietary components, and lifestyle-related factors.

Intervention studies provide a more direct means of testing whether genotype modifies response to a defined dietary exposure. However, even randomized nutritional trials present substantial challenges, including adherence, duration of intervention, heterogeneity in baseline metabolic status, and difficulty in controlling for other lifestyle variables.

Most importantly, demonstration of a genotype × diet interaction within an intervention is not equivalent to demonstration of the clinical utility of genotype-guided nutrition. To establish utility, studies should ideally test whether assignment or adaptation of the dietary intervention on the basis of genotype produces better outcomes than appropriately selected conventional or non-genotype-based personalized recommendations.

This distinction defines a progressively more demanding hierarchy of evidence:

genetic association → functional evidence → replicated gene–diet interaction → prospective prediction → genotype-guided intervention → improved clinical outcome.

**Figure 2:**
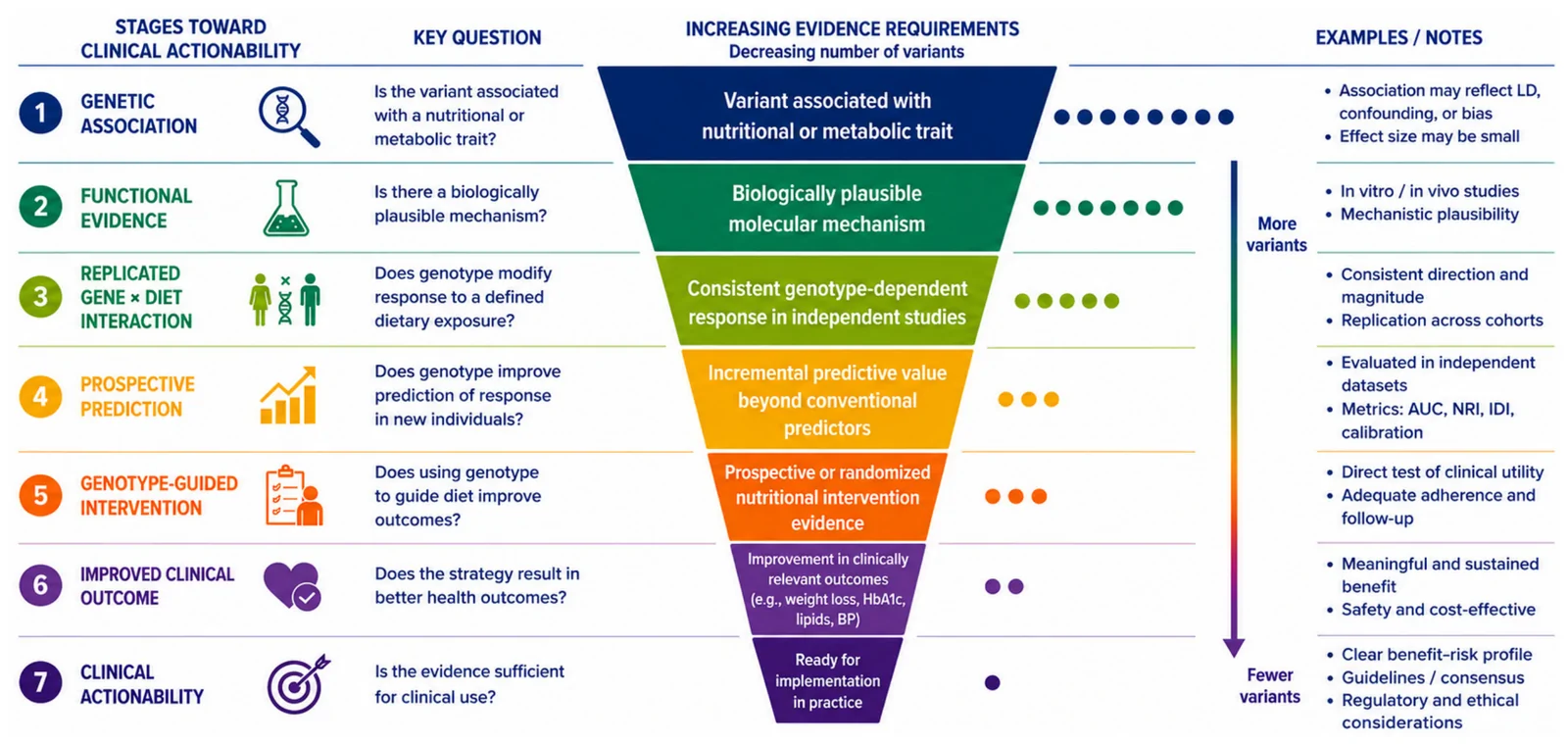
From genetic association to clinical actionability in precision nutrition. Schematic representation of the progressive evidence requirements needed to translate a genetic association into clinically actionable information for precision nutrition. Statistical association represents only the initial level of evidence and should be followed by functional characterization and independent replication of genotype–diet (G × D) interactions. Subsequent levels require demonstration of incremental predictive value in prospective studies, controlled evaluation of genotype-guided nutritional interventions, and evidence of clinically meaningful improvement compared with conventional approaches. The inverted funnel illustrates the progressive reduction in the number of candidate variants expected as increasingly stringent evidence requirements are applied. Clinical actionability represents the final level and requires sufficient evidence to support implementation in practice, together with an overall favorable benefit–risk profile and consideration of relevant regulatory and ethical requirements. This conceptual progression toward clinical actionability is distinct from the seven-domain Weighted Evidence Score (WES) framework used for research-oriented variant prioritization (Section 8.7). LD, Linkage Disequilibrium; AUC, Area Under the Curve; NRI, Net Reclassification Improvement; IDI, Integrated Discrimination Improvement.

Only the later stages of this sequence provide direct evidence that genetic information provides actionable information for precision nutrition. Systematic evaluation of randomized trials incorporating genetic testing into nutrition care has shown that evidence of improvements in clinical outcomes remains weak or insufficient, with substantial heterogeneity among studies and interventions [83,89]. The progressive increase in evidentiary requirements from genetic association to clinical actionability is illustrated in Figure 2.

### 8.5. Predictive Value and Incremental Utility

A genetic marker may have a reproducible biological effect without providing sufficient predictive information to justify its use in individualized nutritional recommendations. Predictive performance therefore needs to be evaluated separately from statistical significance.

The relevant question is not simply whether genotype is associated with response, but whether knowledge of genotype improves prediction beyond information already available from age, sex, anthropometric measurements, family history, baseline metabolic biomarkers, habitual dietary intake, physical activity, and other clinical or behavioral variables.

Genetic information becomes particularly relevant when it provides incremental predictive value over these conventional predictors. This principle also applies to polygenic scores and multivariant panels. Increasing the number of variants does not necessarily improve predictive performance or clinical utility if the additional genomic information contributes little to discrimination, calibration, reclassification, or prediction of intervention response [90].

For the research-oriented genetic panel proposed in the preceding section, candidate variants should therefore ultimately be evaluated not only individually but also according to whether their inclusion improves prediction within integrated models combining genomic, dietary, metabolic, behavioral, and lifestyle information.

### 8.6. Polygenic Models and Multi-Omics Integration

The polygenic architecture of common obesity and related metabolic traits has stimulated the development of polygenic risk scores as a complement to single-variant approaches. Such scores can capture a greater proportion of inherited susceptibility and may help stratify individuals according to genetic predisposition. However, prediction of disease susceptibility and prediction of response to dietary intervention represent fundamentally different objectives [86].

A polygenic score associated with obesity risk does not necessarily identify which dietary intervention will be most effective for an individual. Available evidence for interaction between obesity-related polygenic risk and specific dietary exposures remains mixed, with considerable heterogeneity among studies [85].

More comprehensive models integrating genomic information with transcriptomic, epigenetic, metabolomic, microbiome, and phenotypic data may provide a more realistic representation of individual nutritional responses [91]. However, increased biological resolution is accompanied by greater dimensionality and analytical complexity, together with substantial challenges related to reproducibility and external validation [91]. Multi-omics complexity should therefore not be assumed to confer superior predictive performance unless this is demonstrated in independent datasets and, ultimately, in prospective interventions.

### 8.7. Evidence Framework for Variant Prioritization

To systematically evaluate and prioritize the candidate genetic variants presented in Table 3, we propose a standardized, research-oriented Weighted Evidence Score (WES) integrating seven complementary dimensions of evidence relevant to precision nutrition: biological and functional evidence, phenotypic evidence, independent replication, genotype–diet interaction, intervention evidence, predictive performance, and population validity. Rather than relying solely on statistically significant genetic associations, this framework considers the biological plausibility, reproducibility, nutritional relevance, and potential predictive contribution of each candidate variant.

The seven domains are assigned predefined relative weights, reflecting their contribution to the evaluation of precision nutrition evidence. Functional and phenotypic evidence contribute up to 10 points each; independent replication and predictive value contribute up to 15 points each; genotype × diet interaction and intervention evidence contribute up to 20 points each; and population validity contributes up to 10 points. The resulting WES ranges from 0 to 100 and summarizes the overall strength of evidence supporting the nutritional relevance of a candidate variant.

Within each domain, evidence is graded as strong, moderate, limited, or absent/not demonstrated, corresponding to coefficients of 1, 2/3, 1/3, and 0, respectively. The contribution of each domain is calculated by multiplying its predefined weight by the corresponding coefficient. The overall WES is calculated as the sum of the seven weighted domain contributions. Calculations are performed using unrounded values, and the final WES is reported to one decimal place. The WES is a weighted index of research evidence, rather than a validated measure of clinical utility or the probability of benefit from genotype-guided dietary interventions (Table 4).

For research-oriented prioritization, we propose three provisional evidence tiers based on an equal division of the 0–100 WES scale: Tier 3 (emerging evidence), WES < 100 × 1/3; Tier 2 (moderate evidence), 100 × 1/3 ≤ WES < 100 × 2/3; and Tier 1 (strong evidence), WES ≥ 100 × 2/3, corresponding approximately to thresholds of 33.3 and 66.7 points. These thresholds represent predefined operational boundaries rather than empirically calibrated or clinically validated cutoffs. Tier assignment is based on unrounded WES values and remains subject to additional domain-specific requirements. The three tiers are characterized as follows:

Tier 1—Strong evidence: candidate variants with a high overall WES, at least moderate evidence for formal genotype–diet interactions and prospective nutritional interventions, and pertinent independent replication of the variant-specific diet-related effect. Incremental predictive evidence further strengthens the overall assessment but is not a mandatory requirement for Tier 1 assignment.

Tier 2—Moderate evidence: candidate variants with documented associations with specific nutritional or diet-related phenotypes, supported by human evidence, but with limited, heterogeneous, or incompletely replicated evidence of genotype-dependent responses to nutritional interventions.

Tier 3—Emerging evidence: plausible candidate variant supported by preliminary genetic, molecular, or observational evidence but lacking sufficient independent replication, specific nutritional relevance, or intervention evidence to justify classification in a higher tier.

Importantly, numerical thresholds are necessary but not sufficient for final tier assignment. Tier 2 additionally requires positive variant-specific evidence of nutritional relevance, supported by at least limited evidence of a relevant nutritional phenotype, including dietary perception or preference, food intake, nutrient metabolism, or diet-related physiological or metabolic outcomes. Associations with obesity or disease susceptibility alone are insufficient. Tier 1 additionally requires at least moderate G × D evidence (coefficient ≥ 2/3), at least moderate intervention evidence (coefficient ≥ 2/3), and independent replication supporting the relevant diet-related effect. Replication must concern the specific variant and a sufficiently comparable exposure–outcome relationship rather than a general disease association.

Although positive predictive evidence is not a mandatory criterion for Tier 1 assignment, the substantial weight attributed to predictive value (15% of the total WES) makes the highest tier particularly demanding when incremental predictive performance has not been demonstrated. This reflects the distinction between establishing genotype–phenotype associations or genotype–diet interactions and demonstrating their ability to improve the prediction of individual nutritional responses.

When a candidate reaches a numerical WES threshold but does not satisfy the mandatory requirements for the corresponding tier, it is assigned to the highest lower tier for which all requirements are met. The WES remains unchanged. Thus, the numerical score summarizes the overall evidence, whereas the final tier additionally reflects the minimum requirements for nutritional relevance and evidence maturity.

The proposed domain weights and tier boundaries are heuristic and should be considered provisional, subject to future sensitivity analyses and independent evaluation. The WES–Tier framework is intended to support transparent prioritization and periodic reassessment of candidate variants in research-oriented precision nutrition panels. Domain grades, supporting evidence, relevant null findings, and reasons for tier assignment should be documented to facilitate reproducibility and future updates. Neither a high WES nor Tier 1 classification establishes clinical actionability or demonstrates that genotype-guided dietary recommendations improve health outcomes; such applications require prospective validation and evidence of incremental clinical benefit.

### 8.8. Literature Review and Variant-Specific Evidence Appraisal Methods

#### 8.8.1. Narrative Literature Review

The general literature was synthesized using a narrative review approach. Relevant publications were identified through targeted searches of PubMed/MEDLINE (https://pubmed.ncbi.nlm.nih.gov/, accessed on 26 August 2026 and updated on 10 October 2026) and examination of the reference lists of pertinent studies and reviews. Search concepts included nutrigenetics, nutrigenomics, precision nutrition, gene–diet interactions, food perception and preferences, nutrient metabolism, obesity, and metabolic health. Original studies, reviews, and meta-analyses were considered according to their relevance to the biological and conceptual topics addressed. Selection emphasized scientific relevance, methodological robustness, and the distinction between established findings and emerging or conflicting evidence. This narrative component was not designed as an exhaustive systematic review, and no formal quantitative synthesis was undertaken.

#### 8.8.2. Targeted Literature Assessment of Candidate Genetic Variants

For the twelve variants or haplotypes listed in Table 3, a separate structured appraisal was conducted using variant-specific searches in PubMed/MEDLINE. Gene symbols, rs identifiers, and relevant haplotype names were combined with terms relating to dietary intake, food preferences, nutrient exposure, metabolic phenotypes, genotype–diet interactions, dietary interventions, and predictive performance. Reference lists of relevant publications were examined to identify additional studies. The selection was hypothesis-driven and focused on the nutritional relevance of each candidate variant, rather than on an exhaustive inventory of obesity-associated loci.

Primary human studies were prioritized, particularly independent cohorts, studies reporting formal interaction tests, prospective dietary interventions, and analyses of incremental predictive performance. Functional studies were considered where pertinent to biological plausibility; reviews and meta-analyses were used to contextualize the original evidence. Study protocols without outcome data were not treated as intervention results. Positive, null, and conflicting findings were considered, with attention to variant specificity, dietary exposure and outcome definitions, study design, statistical robustness, multiple testing, population characteristics, and overlap between study cohorts. Findings for a linked variant were not automatically transferred to the candidate under evaluation.

The initial PubMed searches yielded 1185 bibliographic records across the twelve variant-specific searches. Following relevance screening and evidence appraisal according to the criteria described above, 341 records from the initial PubMed searches were retained. During the subsequent locus-by-locus evidence assessment, 31 additional relevant references were progressively identified and incorporated, resulting in 372 variant-specific bibliographic records included in Supplementary Tables S1B–S12B. The numbers of records initially retrieved, retained, and subsequently added for each variant are summarized in Supplementary Table S13A, while the complete bibliographic inventory, distinguishing initial PubMed records from additional references, is provided in Supplementary Table S13B. Because some publications were relevant to more than one variant, these totals represent variant-specific bibliographic records rather than unique publications.

#### 8.8.3. Evidence Grading and Weighted Score Calculation

Each variant was evaluated independently across the seven domains defined in Table 4. Domain grades (strong, moderate, limited, or absent/not demonstrated) were assigned according to the specificity, quality, consistency, and independence of the supporting evidence. The corresponding coefficients (1, 2/3, 1/3, or 0) were multiplied by the predefined domain weights and summed without intermediate rounding; the final WES was reported to one decimal place. The predefined numerical thresholds and mandatory nutritional-evidence requirements described in Section 8.7 were then applied to assign the final Tier. A numerical threshold alone was not sufficient for promotion to a higher Tier.

Evidence grading considers variant specificity, study design, statistical robustness, consistency, independent replication, and the comparability of dietary exposures and outcomes. Evidence concerning a gene or linked genomic region should not automatically be attributed to a specific variant, and multiple publications based on the same study population should not be considered independent replications. The replication domain assesses the reproducibility of relevant findings across independent datasets, whereas the genotype × diet domain specifically evaluates evidence for differential dietary responses.

The genotype × diet interaction (G × D) domain requires evidence that the relationship between a defined dietary exposure and a specified nutritional, metabolic, or clinical outcome differs according to genotype. Positive evidence requires identification of the variant, characterization of dietary exposure and outcome, and a formal statistical test of interaction. Associations between genotype and food preferences, dietary intake, or disease susceptibility do not independently establish G × D interaction. Similarly, genotype-related differences observed during a single dietary intervention, without an appropriate exposure contrast, are insufficient to demonstrate such an interaction. Both observational and intervention studies may contribute to this domain, provided that the interaction is formally evaluated and interpreted in relation to study quality, replication, and consistency.

The intervention-evidence domain is evaluated separately and considers genotype-related responses observed in prospective dietary interventions, with greater weight assigned to well-controlled and replicated findings. Positive results from randomized studies provide stronger support than uncontrolled or exploratory interventions, while null findings and inconsistent results must also be considered. The predictive-value domain evaluates the contribution of genetic information to the prediction of relevant nutritional, sensory, behavioral, biochemical, or metabolic phenotypes and responses, beyond conventional anthropometric, dietary, metabolic, and lifestyle variables. For research-oriented prioritization, limited evidence may include exploratory multivariable models in which the variant-specific contribution is separately quantified, for example through explained variance or an identifiable contribution to model performance, even in the absence of independent validation. Higher evidence levels require progressively stronger demonstrations of incremental predictive performance and model validation. A statistically significant genotype–phenotype association or genotype–diet interaction alone is insufficient to establish predictive value.

Within this research-oriented framework, relevant outcomes are not restricted to body weight, BMI, or clinical disease endpoints, but may also include biologically justified sensory, behavioral, biochemical, metabolic, and physiological phenotypes related to dietary exposure or response. Such evidence may support research prioritization without necessarily demonstrating clinical benefit or the effectiveness of genotype-guided dietary prescriptions. A score of zero in the predictive-value domain indicates that a positive variant-specific predictive contribution has not been sufficiently demonstrated, rather than an absence of biological or scientific relevance. Predictive evidence is evaluated separately from phenotypic associations and genotype–diet interactions to minimize double counting across domains.

To distinguish missing evidence from non-confirmatory findings, each domain may additionally be assigned a documentary status: NE (not evaluated or no pertinent evidence identified), NS (investigated but no supported positive effect demonstrated), MIX (mixed or conflicting evidence), or POS (positive evidence). NE and NS both contribute zero points when no supported positive evidence is available, although they represent different evidentiary situations. Mixed evidence is graded conservatively according to the quality and consistency of the available studies. These documentary descriptors provide qualitative information but do not introduce additional numerical penalties or independently modify the WES.

Variant-specific domain assessments, supporting studies, relevant null or conflicting findings, and methodological limitations are documented in Supplementary Tables S1–S12 (parts A and B for each variant). Supplementary Table S13A summarizes the variant-specific literature screening results, whereas Supplementary Table S13B provides the complete bibliographic inventory, distinguishing records retrieved through the initial PubMed searches from additional references identified during the locus-specific evidence assessment.

### 8.9. Evidence-Based Prioritization of Candidate Genetic Variants

Application of the proposed WES framework to the twelve candidate variants listed in Table 3 resulted in eight variants classified as Tier 2 and four as Tier 3, with none meeting the combined numerical and domain-specific requirements for Tier 1 (Table 5). The distribution of scores reflects substantial differences in the maturity of evidence across functional, phenotypic, and nutritional domains. In particular, well-established biological functions or genotype–phenotype associations do not necessarily translate into reproducible genotype-dependent responses to dietary interventions or improved prediction of individual nutritional outcomes.

The sensory-related variants *TAS2R38* PAV/AVI, *TAS1R2* rs35874116, and *CD36* rs1761667 were primarily supported by evidence concerning taste or fat perception and associated dietary behaviors. *TAS2R38* received the highest WES among these variants because of its well-characterized functional and phenotypic effects, whereas the evidence supporting *TAS1R2* and *CD36* was less extensive or consistent. However, robust genotype–diet interactions and controlled intervention evidence were not sufficiently demonstrated for these variants. Similarly, *FTO* rs9939609, *MC4R* rs17782313, and *LEP* rs7799039 were evaluated in relation to appetite regulation, energy balance, and obesity-related phenotypes. Although the strength of the underlying genetic and phenotypic evidence differs among these variants, reproducible genotype-dependent dietary responses remain insufficiently established, particularly for *MC4R* and *LEP*.

Among variants involved in metabolic regulation, *APOE* ε2/ε3/ε4 showed strong functional and phenotypic evidence, together with moderate support for dietary fat interactions and intervention-related lipid responses. *FADS1* rs174547 and rs174550 were evaluated separately because their evidence bases differ in the strength and consistency of dietary interactions and intervention findings. *PPARG* rs1801282 received substantial support from functional and metabolic evidence and selected dietary intervention studies, although the reproducibility and clinical significance of genotype-dependent responses remain uncertain. Likewise, *TCF7L2* rs7903146 and rs12255372 were assessed independently: rs7903146 has stronger direct functional evidence, whereas both variants have established associations with type 2 diabetes and evidence of dietary interactions, with heterogeneous results across interventions, outcomes, and follow-up periods.

Notably, none of the twelve evaluated variants received a positive score for incremental predictive value. Although exploratory multivariable studies have identified genetic contributions to the prediction of obesity-related phenotypes, including *FTO*, *APOE*, and *PPARG* variants, these findings do not establish that incorporating individual genotypes improves the prediction of dietary intervention responses beyond conventional clinical, anthropometric, and dietary predictors [92]. Similarly, evidence of genotype–diet interactions or genotype-associated treatment responses does not, by itself, demonstrate incremental predictive performance. The uniformly null scores in this domain therefore reflect a specific gap in validated nutrition-related prediction rather than an absence of potentially relevant genetic associations.

Furthermore, differences in study populations, dietary exposures, outcome measures, and statistical approaches, together with incomplete independent replication, limited the strength of several domain-specific assessments. The absence of Tier 1 variants therefore reflects the distinction between biological or nutritional relevance and the more demanding evidence required to support reproducible, genotype-specific dietary recommendations. The resulting classifications are intended to guide research prioritization rather than establish clinical actionability. The studies supporting individual domain assessments, including relevant null or conflicting findings, are documented in Supplementary Tables S1–S12.

### 8.10. Current Limits to Clinical Actionability

Despite rapid progress in nutritional genomics, the evidence required to support routine genotype-guided dietary prescription remains limited for most complex nutritional and metabolic phenotypes. Commercial availability of nutrigenetic testing should therefore not be interpreted as evidence of demonstrated clinical validity or utility [82,83].

Several barriers remain, including small effect sizes of individual variants, incomplete replication of gene–diet interactions, limited ancestral diversity, heterogeneity of dietary assessment and intervention protocols, insufficient prospective validation, and uncertainty regarding the incremental benefit of genomic information over conventional clinical and nutritional variables. Reviews of precision and personalized nutrition interventions suggest that individualized approaches may improve selected dietary or metabolic outcomes in some settings, but evidence remains heterogeneous and does not establish that genomic information is the component responsible for the observed benefit [93].

These limitations do not diminish the value of nutrigenetic research. Rather, they define the experimental requirements necessary for its translation. Well-designed prospective studies integrating standardized genetic information with accurately measured dietary exposure, intermediate molecular phenotypes, and clinically relevant outcomes will be essential to determine which genetic markers can progress from association to prediction and, ultimately, to actionability.

Precision nutrition should therefore be regarded as a developing evidence-based framework rather than as a direct consequence of genomic testing. The central challenge is not merely to identify genetic differences among individuals, but to demonstrate when those differences provide reproducible, incremental, and clinically meaningful information capable of improving nutritional interventions.

## 9. Ethical and Social Challenges in Precision Nutrition

The increasing integration of genomic information into nutritional research raises ethical and social issues that extend beyond the analytical validity of genetic testing. Unlike many conventional nutritional biomarkers, germline genetic information is relatively stable throughout life, may reveal information beyond the original purpose of testing, and can have implications not only for the individual tested but also for biologically related family members. These characteristics require particular caution when genetic data are generated, interpreted, or communicated in the context of precision nutrition [94,95].

These issues become especially relevant when research-oriented genetic profiling is considered for clinical or commercial use. The availability of genetic information does not itself establish clinical utility, and premature implementation may expose individuals to risks without demonstrated nutritional benefits [94].

### 9.1. Informed Consent and Incidental Genetic Information

Genetic analyses performed for nutritional purposes may generate information whose significance extends beyond nutrition. Depending on the genes and genomic regions analyzed, testing may reveal variants associated with disease susceptibility, carrier status, pharmacogenetic traits, or other medically relevant phenotypes that were not the primary objective of the analysis.

This possibility is particularly important when sequencing-based approaches interrogate entire genes, broad genomic regions, or exomes and genomes rather than a predefined set of variants. A distinction should therefore be made between incidental findings, which emerge unintentionally during analysis, and secondary findings, which are deliberately sought despite being unrelated to the primary indication for testing [96]. The distinction has direct implications for the research-oriented panel proposed in this Review: targeted genotyping of selected nutritionally relevant variants presents a substantially different informational and ethical context from that of sequencing genes that may contain additional variants with established clinical significance.

Participants should therefore be adequately informed about the scope of the genetic analysis, the possibility of incidental or secondary findings, and whether such information will be actively searched for, interpreted, or returned. Policies concerning the right to know and the right not to know should be defined before testing and should clearly distinguish exploratory research findings from clinically validated information. Informed consent should be understood as a process enabling participants to appreciate the nature and potential implications of testing rather than simply as the completion of a consent form [96].

The inclusion of genes with well-established implications outside nutrition deserves particular consideration. *APOE* provides a paradigmatic example. Determination of *APOE* genotype for investigating lipid metabolism or dietary response may simultaneously provide information associated with susceptibility to other health conditions. Thus, even when the nutritional research question concerns lipid response, disclosure of the genotype may convey information whose implications extend substantially beyond nutrition. The scientific rationale for including such markers should therefore be weighed against the additional responsibilities associated with interpretation, consent, communication, and possible disclosure.

### 9.2. Privacy, Data Protection, and Familial Implications

Genomic data differ from many other health-related data because they are persistent, highly individual, and partially shared among biological relatives. Genetic information generated for precision nutrition research can therefore have implications for individuals who have not themselves undergone testing. Ethical considerations surrounding genetic information consequently extend beyond individual privacy to questions concerning familial interests, confidentiality, and communication.

Secure storage, controlled access, appropriate governance, and transparent policies for data sharing are essential when genomic information is combined with detailed dietary, metabolic, behavioral, and clinical data. The increasing use of multi-omics datasets further amplifies these concerns because integration of multiple biological and phenotypic layers increases the informational richness of individual profiles and may increase challenges related to data protection, re-identification risk, and confidentiality.

These considerations should be incorporated into the design of precision nutrition studies rather than addressed only after data generation. Participants should understand how their genomic and phenotypic information will be stored, analyzed, shared, and potentially reused, particularly when long-term repositories, data sharing, or secondary research uses are envisaged. The European Society of Human Genetics has similarly emphasized privacy, confidentiality, secure data handling, and transparency regarding subsequent research uses in the context of genetic testing [94].

Familial implications should nevertheless be interpreted proportionately. The ethical significance of information relevant to relatives depends on the magnitude and seriousness of the associated risk and on whether effective preventive or therapeutic measures are available. Recent European Society of Human Genetics (ESHG) recommendations similarly emphasize that approaches to communicating genetic risk within families should reflect the balance between potential benefit and harm and the actionability of the finding [94].

### 9.3. Genetic Determinism, Communication, and Commercial Testing

One of the principal risks associated with nutrigenetic information is genetic determinism. Complex nutritional and metabolic phenotypes arise from multiple genetic, biological, behavioral, and environmental influences. Accordingly, variants associated with food preferences, obesity susceptibility, or metabolic traits should be communicated as probabilistic contributors rather than determinants of individual nutritional requirements or future health outcomes.

This distinction is especially important in the context of commercially available and direct-to-consumer nutrigenetic tests, where the technical ability to genotype a variant may precede convincing evidence of predictive or clinical utility. As discussed in the previous section, association and biological plausibility do not necessarily imply actionability. Professional and scientific organizations have consequently emphasized clinical validity and utility, transparent communication, appropriate pre-test information, privacy protection, and access to qualified professional advice as important requirements for responsible genetic testing [94,97].

Communication of genetic results also requires explicit attention to uncertainty. Relative effects, probabilistic associations, population-specific evidence, and limitations of prediction may be difficult to translate into information that is both understandable and scientifically accurate. Genetic information should support informed decision-making rather than create unwarranted perceptions of biological inevitability or false precision.

Professional responsibility therefore represents an important component of nutrigenetic implementation. Interpretation of genomic information requires competencies extending beyond conventional dietary counseling, particularly when findings have potential health implications outside nutrition. Appropriate education of nutrition professionals, clear boundaries of professional competence, and access to genetics expertise or genetic counselling when indicated will be important if nutrigenetic information moves from research settings toward clinical practice [98].

### 9.4. Equity, Population Diversity, and Access to Precision Nutrition

Precision nutrition also raises broader questions of equity and representativeness. Genetic associations and predictive models derived predominantly from populations of limited ancestral diversity may not perform equally across populations because of differences in allele frequencies, linkage disequilibrium patterns, environmental exposures, dietary practices, and socioeconomic contexts. The lack of diversity in genomic research is therefore not merely a methodological limitation but also an ethical concern when predictive tools developed in one population are proposed for broader implementation.

The problem extends beyond genomic diversity. Access to sequencing, molecular profiling, specialized nutritional counselling, and longitudinal monitoring may be unevenly distributed. A precision nutrition strategy requiring extensive genomic and multi-omics characterization could therefore increase existing disparities if its benefits become available primarily to individuals or healthcare systems with greater resources.

Furthermore, dietary behavior cannot be understood solely through biological information. Food availability, cultural practices, education, socioeconomic conditions, family environment, and access to healthy dietary options strongly influence what individuals are realistically able to consume. A genetically informed recommendation that is biologically plausible but economically, culturally, or practically inaccessible cannot constitute effective precision nutrition.

Equity should therefore be considered at two complementary levels: equity in the generation and interpretation of genomic evidence, ensuring adequate representation of diverse populations, and equity in the implementation of nutritional recommendations, ensuring that personalization does not ignore the social and material conditions that determine dietary choice.

Responsible precision nutrition must therefore combine scientifically validated approaches with appropriate genomic-data governance, professional interpretation, transparent communication, and equitable access, while accounting for the social and environmental conditions that shape dietary choices.

**Figure 3:**
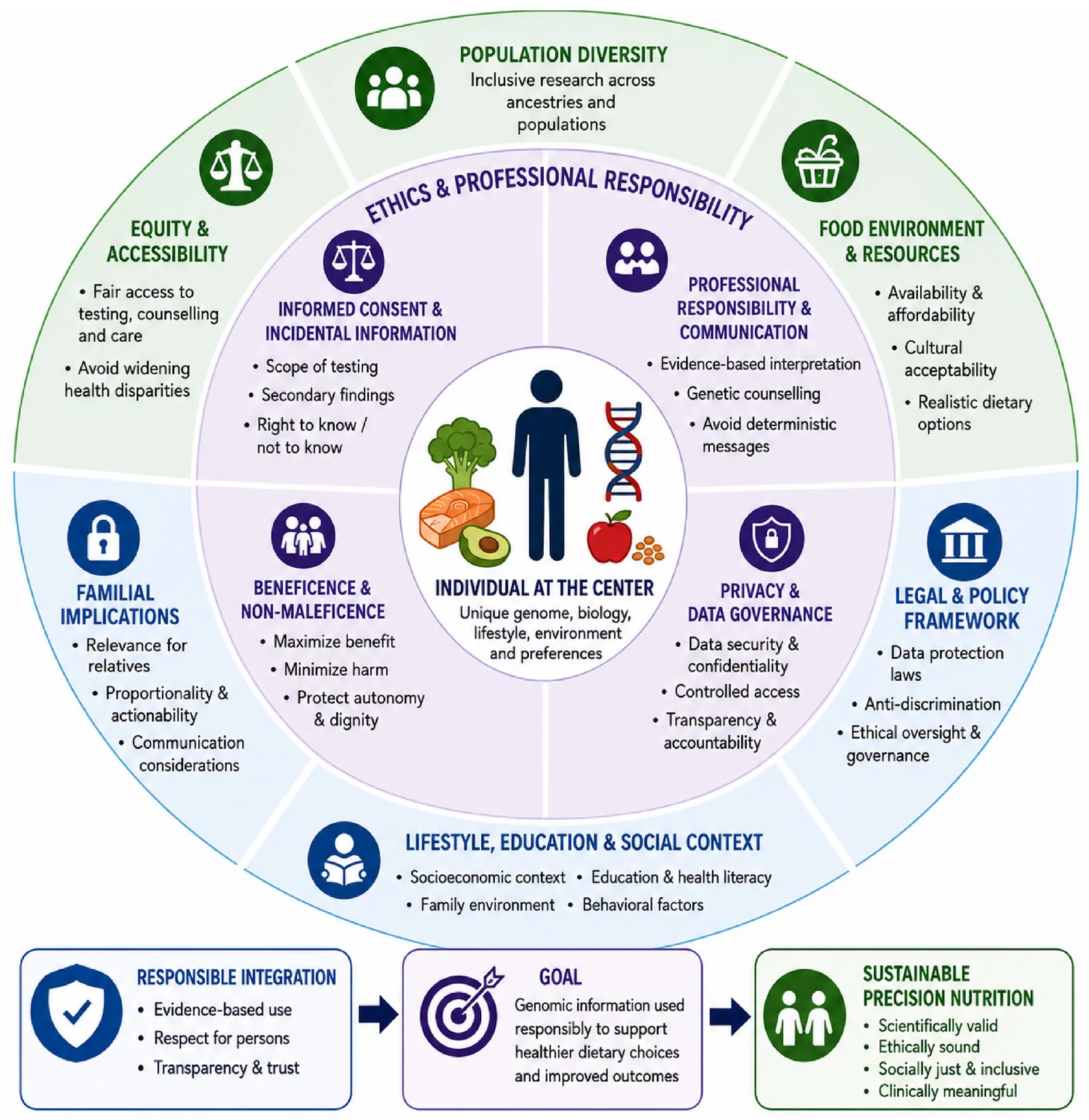
Ethical, professional, and social dimensions of precision nutrition. Schematic representation of the major ethical, professional, and social considerations associated with the integration of genomic information into precision nutrition. The individual is placed at the center of the framework, surrounded by ethical and professional responsibilities directly related to the generation, interpretation, and use of genomic information, including informed consent and the management of incidental or secondary findings, professional responsibility and communication, privacy and data governance, and the principles of beneficence and non-maleficence. These dimensions are embedded within a broader social and environmental context encompassing population diversity, equity and accessibility, food environment and resources, legal and policy frameworks, familial implications, and socioeconomic, educational, and lifestyle factors. Responsible integration across these interconnected levels is essential to ensure that genomic information is used in an evidence-based, transparent, and proportionate manner, with the ultimate goal of developing responsible precision nutrition strategies that are scientifically valid, ethically sound, socially equitable and inclusive, and clinically meaningful.

These interconnected biological, ethical, professional, and social dimensions of precision nutrition are summarized in Figure 3.

## 10. Future Perspectives and Conclusions

Nutrigenetics and nutrigenomics have substantially expanded our understanding of the complex relationships linking genetic variability, dietary exposure, molecular responses, and metabolic phenotypes. However, the translation of this knowledge into effective precision nutrition strategies remains an evolving objective rather than an established clinical reality. The major challenge is no longer simply to identify additional genetic associations, but to determine which components of interindividual variability are biologically meaningful, reproducible, predictive, and ultimately actionable [5,82].

A central contribution of this review is the integration of genetically influenced dietary exposure and genotype-dependent biological responses within a unified diet–genome–phenotype framework. This perspective connects relatively stable genetic determinants with dynamic molecular, microbial, behavioral, and environmental influences, providing a basis for investigating interindividual variability in nutritional responses.

### 10.1. From Association to Prospective Validation

Future studies should increasingly move from retrospective association toward prospective, hypothesis-driven validation. Candidate gene–diet interactions should be evaluated using accurately characterized dietary exposures, functionally relevant genetic variants, intermediate molecular and metabolic phenotypes, and predefined clinical or physiological outcomes. Wherever possible, the variant, dietary exposure, direction of the expected interaction, and primary outcome should be specified a priori, reducing the risks associated with multiple testing and exploratory subgroup analyses. Replication across independent populations representing diverse ancestral and geographic backgrounds will be essential, as will harmonization of dietary assessment, phenotypic characterization, and analytical approaches.

Longitudinal nutritional intervention studies will be particularly important for establishing predictive value. Rather than asking only whether a genetic variant is associated with a metabolic phenotype, future investigations should determine whether genotype predicts response to a defined dietary intervention and whether incorporation of genetic information improves prediction beyond established clinical, anthropometric, metabolic, behavioral, and lifestyle variables. Ultimately, the most compelling evidence for actionability will require demonstrating that genetically informed nutritional strategies produce meaningful benefits compared with appropriately selected conventional approaches [83,89].

The pilot WES application to twelve illustrative variants highlights the limited predictive evidence currently available for genotype-informed nutritional interventions. None of the evaluated variants met the proposed criteria for incremental predictive value, emphasizing the need for independently validated models that integrate genetic information with dietary, metabolic, and other relevant individual-level variables.

### 10.2. Multi-Omics Integration and Computational Modeling

The integration of genomic data with other molecular layers offers an additional opportunity. Epigenomic, transcriptomic, metabolomic, and microbiome profiles may provide dynamic information on biological state and response that complements the relatively stable information encoded by germline variation [91]. Advances in computational modeling and artificial intelligence may facilitate the integration of these heterogeneous datasets and the identification of multidimensional response patterns that cannot be captured by individual biomarkers [99]. Such approaches could ultimately support the transition from static risk estimation toward dynamic prediction of nutritional response. However, increasing model complexity should not be equated with improved predictive performance or clinical utility. Transparency, reproducibility, interpretability, external validation, and demonstration of incremental predictive value will remain essential requirements [99].

### 10.3. Standardization, Predictive Value, and Incremental Utility

A further priority is the prospective evaluation and refinement of standardized frameworks for genetic-marker prioritization. The research-oriented WES approach proposed in this review provides a structured basis for comparing candidate variants across complementary evidence domains, while allowing their classification to be updated as new functional, intervention, and predictive evidence becomes available. Its value will depend on reproducibility, independent validation, and the ability to identify markers that contribute meaningful information to nutritional research.

The future of precision nutrition is also unlikely to depend on genomic information alone. The relative contribution of genetics should be evaluated against—and integrated with—variables that are easier and less costly to obtain, including dietary intake, anthropometric measurements, metabolic biomarkers, physical activity, behavioral characteristics, and socioeconomic context. A genomic or multi-omics model should therefore be considered advantageous only when it provides reproducible information beyond what can be obtained from simpler approaches. This principle of incremental utility is essential to prevent technological sophistication from becoming an objective in itself.

### 10.4. Responsible Translation and Future Directions

Technological feasibility alone should not determine clinical implementation. The use of genomic and multi-omics information must remain proportionate to its demonstrated scientific and clinical value and be accompanied by appropriate safeguards for informed consent, privacy, communication, professional interpretation, and equitable access. These requirements are particularly important when genetic findings have implications extending beyond nutrition.

Precision nutrition should therefore not be viewed as a replacement for population-based nutritional recommendations. Population-level evidence will continue to provide the foundation of nutritional guidance, whereas precision approaches may offer an additional level of stratification when reproducible interindividual differences can be demonstrated to influence response to intervention. The relevant question is consequently not whether nutrition can become completely individualized, but when personalization provides a measurable advantage over well-established population-based recommendations.

Progress toward this objective will require the convergence of molecular biology, human genetics, nutrition science, behavioral research, well-designed intervention studies, and rigorous predictive validation. The ultimate goal of precision nutrition should not be to generate increasingly detailed molecular profiles of individuals, but to identify the smallest and most informative combination of genomic, molecular, phenotypic, and contextual variables capable of improving nutritional decisions. Precision should therefore be defined not by the amount of information collected, but by the extent to which that information leads to nutritional interventions that are demonstrably more predictive, effective, safe, equitable, and appropriate for the individual.

## Figures and Tables

**Table 1:** Representative genetic determinants of food perception, eating behavior, and dietary exposure.

Gene/Locus	Representative Variant(s)	Primary Trait	Potential Effect on Dietary Exposure	Strength/Consistency of Evidence	Ref. (a)
*TAS2R38*	rs713598, rs1726866, rs10246939	Bitter taste	Bitter-food acceptance and intake	Strong for PTC/PROP perception; weaker for habitual intake	[4,6]
*TAS1R2*	rs35874116	Sweet taste perception	Sweet-food preference and sugar intake	Moderate; variable across populations and studies	[6,12]
*TAS1R3*	rs307355, rs35744813	Sweet taste sensitivity	Sweet-food preference and sugar intake	Strong for sucrose sensitivity; limited for habitual intake	[6,21]
*CD36*	rs1761667	Oral fat perception	Fat-rich food preference and intake	Relatively strong for fat perception; less consistent for habitual intake	[13,14]
*FTO*	rs9939609	Appetite and eating behavior	Energy intake and food choice	Evidence for appetite/eating-related traits and energy-dense food choice; consistency varies across studies	[22,23]
*MC4R*	rs17782313	Appetite and satiety	Energy intake and eating behavior	Stronger for appetite-related traits than measured energy intake	[20]

Note: (a), representative references. PTC/PROP, Phenylthiocarbamide/6-n-propylthiouracil.

**Table 2:** Representative examples of diet-sensitive epigenetic mechanisms relevant to nutrigenomics and precision nutrition.

Dietary/Metabolic Exposure	Epigenetic Mechanism	Representative Molecular Link	Human/Experimental Evidence	Main Interpretative Limitation	Ref.
Folate and other one-carbon nutrients	DNA methylation	SAM availability; DNMT activity; *MTHFR* C677T	Genotype-dependent relationship between folate status/intake and DNA methylation	Tissue- and genomic-context dependence; causality	[41,42]
Developmental undernutrition	DNA methylation	*IGF2* DMR	Dutch Hunger Winter	Association does not establish phenotypic mediation	[34]
Sulforaphane/cruciferous-derived isothiocyanates	Histone regulation	HDAC inhibition	Mechanistic and human intervention evidence	Dose, bioavailability and tissue exposure	[27,48]
Dietary interventions	Non-coding RNA regulation	Diet-responsive miRNAs	Changes in circulating/tissue miRNA profiles following dietary interventions	Heterogeneity and poor standardization	[47]

Note: SAM, *S-adenosylmethionine*; DNMT, DNA methyltransferase; *MTHFR*, methylenetetrahydrofolate reductase; *IGF2*, insulin-like growth factor 2; DMR, differentially methylated region; HDAC, histone deacetylase; miRNA, microRNA.

**Table 3:** Candidate genetic variants for a research-oriented precision nutrition panel in overweight and obesity.

Functional Domain	Gene/ Locus	Candidate Variant(s)	Biological/Nutritional Rationale	Nutritional Phenotype and Evidence of Genotype–Diet Interaction, Where Available	Ref. (a)
Bitter taste/food perception	*TAS2R38*	rs713598; rs1726866; rs10246939	Functional determinants of the major PAV/AVI bitter-taste haplotypes	Bitter-food perception, acceptance, and intake	[4,6]
Sweet taste/food perception	*TAS1R2*	rs35874116	Sweet-taste receptor function	Sweet perception, sugar preference/intake and dietary quality	[12,73]
Oral fat perception	*CD36*	rs1761667	Fatty-acid sensing and oral fat sensitivity	Fat perception, preference, and intake	[13,74]
Appetite/energy balance	*FTO*	rs9939609	Appetite/eating behavior and obesity susceptibility	Energy intake/eating behavior; limited evidence for genotype-dependent weight-loss response	[17,22]
Appetite/satiety	*MC4R*	rs17782313	Central melanocortin signaling and appetite regulation	Appetite and potential energy/macronutrient intake	[20]
Appetite/energy homeostasis	*LEP*	rs7799039	Leptin signaling and regulation of energy homeostasis	Exploratory associations with metabolic responses to energy-restricted dietary interventions	[75,76]
Lipid metabolism	*APOE*	rs429358 + rs7412	Define ε2/ε3/ε4 isoforms; lipoprotein metabolism	Dietary fat-dependent lipid responses	[77,78]
PUFA metabolism	*FADS1*	rs174547	Endogenous PUFA desaturation	Linoleic acid × genotype effects on lipid/adiposity traits	[29,79]
PUFA metabolism	*FADS1*	rs174550	Endogenous PUFA desaturation	Genotype-dependent response to LA- and ALA-enriched diets	[31]
Adipocyte/metabolic regulation	*PPARG*	rs1801282 (Pro12Ala)	Adipogenesis, insulin sensitivity and lipid metabolism	Dietary fat × genotype effects on metabolic/lipid traits	[66,80]
Glucose metabolism	*TCF7L2*	rs7903146	Glucose homeostasis/metabolic response	Genotype-dependent responses to dietary/lifestyle interventions across multiple RCTs	[67,68]
Glucose metabolism	*TCF7L2*	rs12255372	Glucose homeostasis/body-composition response	Dietary fat × genotype effects on adiposity and glycemic outcomes; limited replication	[65]

Note: (a) representative references. PAV, proline–alanine–valine; AVI, alanine–valine–isoleucine; PUFA, polyunsaturated fatty acid(s); LA, linoleic acid; ALA, α-linolenic acid; RCTs, randomized controlled trials. The evidence summarized in this table encompasses distinct levels of nutritional relevance, including sensory and behavioral phenotypes, genetic susceptibility, metabolic associations, and genotype-dependent responses to dietary exposures or interventions. The inclusion of a variant does not imply that a genotype–diet interaction has been demonstrated. The strength and nature of the supporting evidence are evaluated separately in Section 8.6, Section 8.7 and Section 8.8.

**Table 4:** Proposed Weighted Evidence Score (WES) framework for candidate genetic variants in precision nutrition research.

Evidence Domain	Max Points	Strong (a)	Moderate (a)	Limited (a)
Functional evidence	10	Direct, well-supported variant-specific functional evidence	Functional evidence with methodological or replication limitations	Preliminary or indirect mechanistic evidence
Phenotypic evidence	10	Robust, well-characterized association with relevant nutritional or metabolic phenotypes	Supported association with some methodological limitations or heterogeneity	Preliminary or weakly characterized association
Independent replication	15	Consistent findings across multiple independent studies	Partial independent replication with some limitations	Limited independent replication, with substantial methodological or consistency limitations
Genotype × diet interaction	20	Robust, independently replicated formal interactions	Independently supported interactions with some limitations or heterogeneity	Isolated or exploratory formal interaction
Intervention evidence	20	Robust, replicated genotype-specific responses in controlled dietary interventions	Positive prospective intervention evidence with limitations	Preliminary or uncontrolled intervention evidence
Incremental predictive value	15	Robust, independently validated incremental prediction beyond conventional variables	Quantitatively demonstrated incremental predictive improvement, with internal or external validation but methodological limitations	Exploratory variant-specific predictive contribution quantified in a relevant multivariable model, without requiring independent validation
Population validity	10	Consistent evidence across multiple populations or ancestries	Evidence from more than one population with limited transferability assessment	Evidence restricted to a single population or setting
Total	100			

Note. (a) Evidence is graded as strong (coefficient 1), moderate (2/3), limited (1/3), or absent/not demonstrated (0), relative to the maximum points assigned to each domain. The contribution of each domain is calculated by multiplying its maximum weight by the corresponding coefficient. For independent replication, a single study does not constitute replication, and multiple publications from the same cohort are not counted as independent evidence. For predictive value, limited evidence may include a separately quantified genotype contribution in an exploratory multivariable model, whereas moderate and strong evidence require progressively more robust demonstrations of incremental predictive performance. Evidence not evaluated or not sufficiently demonstrated receives zero points. The WES is the sum of the seven weighted contributions, calculated without intermediate rounding.

**Table 5:** Application of the Weighted Evidence Score (WES) to candidate genetic variants for precision nutrition research.

Gene/Variant	F	P	R	G × D	I	V	Pop	WES	Tier
Max Points	10	10	15	20	20	15	10	100	
*TAS2R38* PAV/AVI	S (10.0)	S (10.0)	M (10.0)	0	0	0	M (6.7)	36.7	2
*TAS1R2* rs35874116	M (6.7)	M (6.7)	L (5.0)	0	0	0	L (3.3)	21.7	3
*CD36* rs1761667	M (6.7)	M (6.7)	M (10.0)	0	0	0	L (3.3)	26.7	3
*FTO* rs9939609	M (6.7)	S (10.0)	M (10.0)	L (6.7)	L (6.7)	0	M (6.7)	46.7	2
*MC4R* rs17782313	L (3.3)	L (3.3)	0	L (6.7)	0	0	M (6.7)	20.0	3
*LEP* rs7799039	L (3.3)	L (3.3)	0	0	L (6.7)	0	L (3.3)	16.7	3
*APOE* ε2/ε3/ε4	S (10.0)	S (10.0)	M (10.0)	M (13.3)	M (13.3)	0	M (6.7)	63.3	2
*FADS1* rs174547	M (6.7)	S (10.0)	M (10.0)	M (13.3)	L (6.7)	0	M (6.7)	53.3	2
*FADS1* rs174550	M (6.7)	S (10.0)	M (10.0)	M (13.3)	M (13.3)	0	L (3.3)	56.7	2
*PPARG* rs1801282	S (10.0)	S (10.0)	M (10.0)	M (13.3)	M (13.3)	0	L (3.3)	60.0	2
*TCF7L2* rs7903146	S (10.0)	S (10.0)	M (10.0)	M (13.3)	M (13.3)	0	L (3.3)	60.0	2
*TCF7L2* rs12255372	L (3.3)	S (10.0)	M (10.0)	M (13.3)	M (13.3)	0	L (3.3)	53.3	2

Note: PAV, proline–alanine–valine; AVI, alanine–valine–isoleucine; F, functional evidence; P, phenotypic evidence; R, independent replication; G × D, genotype–diet interaction; I, intervention dietary; V, incremental predictive value; Pop, population validity; WES, Weighted Evidence Score. S, strong; M, moderate; L, limited; 0, absent/not demonstrated. Values in parentheses indicate weighted domain scores. The supporting evidence, relevant null or conflicting findings, and domain-specific justifications are provided in Supplementary Tables S1–S12.

## Data Availability

No new experimental data were generated in this study. The evidence assessments and Weighted Evidence Score (WES) calculations are reported in the manuscript and accompanying Supplementary Materials.

## Supplementary Materials

The following supporting information can be downloaded at https://www.mdpi.com/article/10.3390/nu18213486/s1: Tables S1–S12: Variant-specific evidence assessment and Weighted Evidence Score (WES) evaluation for the 12 genetic loci/variants examined in this review. For each variant, Part A (Tables S1A–S12A) provides the domain-specific WES assessment, supporting rationale, and overall evidence classification, whereas Part B (Tables S1B–S12B) presents the supporting bibliographic evidence, including study-level assessments and evidence annotations (POS, NEG, and NEU). Table S13: Documentation of the literature search and evidence inventory. Table S13A summarizes the variant-specific literature searches, screening procedures, and study selection counts. Table S13B provides the complete bibliographic inventory, including the initially retrieved PubMed records and additional references identified during the variant-specific evidence assessment, with information on their provenance and inclusion status.

## Abbreviations

The following abbreviations are used in this manuscript:

AMPK	AMP-activated protein kinase
APOE	Apolipoprotein E
BMI	Body mass index
CD36	Cluster of differentiation 36
ChREBP	Carbohydrate-responsive element-binding protein
DMR	Differentially methylated region
DNMT	DNA methyltransferase
FADS1	Fatty acid desaturase 1
FADS2	Fatty acid desaturase 2
FTO	Fat mass and obesity-associated gene
FXR	Farnesoid X receptor
HDAC	Histone deacetylase
IGF2	Insulin-like growth factor 2
KEAP1	Kelch-like ECH-associated protein 1
LXR	Liver X receptor
MC4R	Melanocortin 4 receptor
mTORC1	Mechanistic target of rapamycin complex 1
MTHFR	Methylenetetrahydrofolate reductase
NAD^+^	Nicotinamide adenine dinucleotide
NF-κB	Nuclear factor kappa B
NRF2	Nuclear factor erythroid 2-related factor 2
PPAR	Peroxisome proliferator-activated receptor
PPARG	Peroxisome proliferator-activated receptor gamma
PROP	6-n-propylthiouracil
PTC	Phenylthiocarbamide
PUFA	Polyunsaturated fatty acid
RCT	Randomized controlled trial
SAM	S-adenosylmethionine
SCFA	Short-chain fatty acid
SREBP	Sterol regulatory element-binding protein
TAS1R2	Taste receptor type 1 member 2
TAS1R3	Taste receptor type 1 member 3
TAS2R38	Taste receptor type 2 member 38
TCF7L2	Transcription factor 7-like 2
